# Biotransformation of Maclekarpine E in Rats: CYP2C19-Mediated Metabolism, Fecal Enrichment, and Network Pharmacology-Based Anti-Ulcerative Colitis Prediction

**DOI:** 10.3390/cimb48030335

**Published:** 2026-03-23

**Authors:** Yingxue Yang, Lin Wang, Jiaojiao Xue, Zhen Dong, Pi Cheng

**Affiliations:** 1 Hunan Province Key Laboratory of Traditional Chinese Veterinary Medicine, Hunan Agricultural University, Changsha 410128, China; 1879349968@stu.hunau.edu.cn (Y.Y.); wl92606@126.com (L.W.); x15065441211@163.com (J.X.); 2 Chinese Medicinal Materials Breeding Innovation Centre of Yuelushan Laboratory, Changsha 410128, China; 3 Shiyan Key Laboratory of Biological Resources and Eco-Environmental Protection, College of Chemical and Environmental Engineering, Hanjiang Normal University, Shiyan 442000, China

**Keywords:** maclekarpine E, metabolite identification, CYP2C19, ABCG2, network pharmacology

## Abstract

Maclekarpine E is a minor alkaloid from *Macleaya* species with reported in vitro anti-inflammatory activity, but its in vivo metabolism remains unexplored. This study investigated the metabolic fate of maclekarpine E in rats and evaluated the potential pharmacological relevance of its metabolites. Maclekarpine E was orally administered to male Sprague-Dawley rats (250 mg/kg). Plasma, urine and feces were collected and analyzed by UPLC-Q-TOF-MS/MS. CYP phenotyping was performed using recombinant human enzymes. Molecular docking against ABCG2 and ABCC2 was conducted to assess potential interactions of all fecal compounds with these efflux transporters. Network pharmacology was employed to predict potential anti-ulcerative colitis-related targets of the metabolites, generating hypotheses for future experimental validation. Nineteen phase I metabolites were identified. Biotransformations included ring-opening, demethylation and oxidation. All 19 metabolites were detected in feces, nine in plasma and two in urine. No phase II conjugates were observed. CYP2C19 was the only significantly active isoform under the tested conditions, mediating approximately 16.5% substrate depletion (*p* < 0.05). All 20 fecal compounds bound ABCG2 (ΔG < −5.0 kcal/mol); 19 bound ABCC2. Network pharmacology yielded 57 overlapping targets with ulcerative colitis, enriched in PI3K-Akt and MAPK pathways. This study provides the first comprehensive metabolic profile of maclekarpine E in rats. The compound undergoes CYP2C19-mediated oxidation and is predominantly excreted into feces. Its fecal metabolites are potential ABCG2/ABCC2 substrates and may target UC-associated pathways based on network pharmacology predictions, warranting further experimental validation.

## 1. Introduction

The genus Macleaya comprises two main species, *Macleaya cordata* and *Macleaya microcarpa*, which are known to be rich in isoquinoline alkaloids and have traditionally been used in Chinese medicine, albeit primarily for external applications [1]. Phytochemical and pharmacological investigations over recent decades have predominantly concentrated on the major alkaloids—most notably sanguinarine and chelerythrine. These compounds have been extensively characterized for their anti-inflammatory [2], anticancer, and growth-promoting properties [3], leading to their successful translation into veterinary feed additives and investigational human therapeutics. Despite these advances, the exploitation of *Macleaya* alkaloids remains incomplete; current research efforts have overwhelmingly prioritized high-abundance constituents, whereas the minor alkaloids have attracted far less attention.

Maclekarpine E is a dihydrosanguinarine derivative and represents one such minor constituent in both *Macleaya* species. Structurally, it is distinguished by a feruloyl moiety at the C-12 position—a unique side chain absent in the major alkaloids sanguinarine and chelerythrine. Recent investigations into plant alkaloids have increasingly recognized that minor constituents can possess both unprecedented chemical architectures and pharmacological profiles distinct from their more abundant counterparts. For instance, the trace hetero-dimeric alkaloids yanhusamides A-C from *Corydalis yanhusuo* feature a novel benzylisoquinoline–pyrrole hybrid skeleton [4], while datumetine, a minor alkaloid from Datura species, exhibits unique NMDA receptor modulatory effects not shared by major tropane alkaloids [5]. These examples underscore the potential pharmacological value of investigating trace constituents. Despite this growing recognition, the pharmacological characterization of maclekarpine E has, to date, the pharmacological characterization of maclekarpine E has been limited to a small number of in vitro studies, which have reported modest anti-inflammatory [6] and antiproliferative activities [7] in cultured cell lines. Whether these preliminary observations possess any in vivo relevance has remained entirely unknown—until recently. Our research group has completed an unpublished murine study demonstrating that oral administration of maclekarpine E significantly ameliorates dextran sulfate sodium-induced colitis, providing the first in vivo evidence that this trace alkaloid exerts therapeutic efficacy against ulcerative colitis (UC), a chronic and relapsing inflammatory disorder of the colorectum with limited curative options [8].

This raises a fundamental question: whether the observed anti-colitic effect is attributable to the parent compound alone or also involves its metabolites, which may exhibit distinct or enhanced pharmacological activities. For orally administered phytochemicals, the answer frequently lies not only in the parent compound but also in its metabolic products [9,10]. Gastrointestinal stability determines the fraction available for absorption [11]; hepatic cytochrome P450 (CYP)-mediated metabolism governs systemic exposure and clearance, and, importantly, metabolites themselves may retain or acquire biological activities distinct from those of the parent molecule. In the context of UC, where the colonic mucosa is both the pathological target and a direct interface with intestinal contents, metabolites excreted into the fecal stream may represent particularly relevant effectors acting locally at the diseased tissue. However, the metabolic fate of maclekarpine E has never been systematically investigated. Its oral stability, its metabolite profile across biological matrices, and the specific CYP isoforms contributing to its biotransformation remain entirely uncharacterized.

We, therefore, hypothesized that the anti-colitic efficacy of maclekarpine E may be partially attributable to its metabolites, and that the distinctive metabolite profile generated during its in vivo disposition—particularly those products selectively enriched or uniquely present in feces—may operate through mechanisms complementary to, or distinct from, the parent compound. To test this hypothesis, the present study was designed to provide the first comprehensive characterization of maclekarpine E metabolism in rats. Using high-resolution quadrupole time-of-flight mass spectrometry, we sought to identify and compare metabolites present in plasma, urine, and feces following oral administration. Artificial gastric and intestinal fluid models were employed to assess oral stability, and a panel of recombinant human CYP enzymes (1A2, 2C9, 2D6, 3A4, and 2C19) was utilized to evaluate the contribution of major metabolic isoforms. Furthermore, to explore the potential pharmacological relevance of fecal metabolites, a network pharmacology approach was integrated to predict their putative UC-related targets and signaling pathways. By integrating conventional metabolism study with computational target prediction, this work aims to bridge the gap between the observed in vivo efficacy of maclekarpine E and its active molecular species, thereby establishing a metabolite-oriented foundation for future mechanism-driven drug development in the treatment of ulcerative colitis.

## 2. Materials and Methods

### 2.1. Materials

Maclekarpine E was chemically synthesized in our laboratory (purity ≥ 98%) [6]. Recombinant human CYP isoforms (CYP1A2, CYP2C9, CYP2C19, CYP2D6, and CYP3A4), an NADPH-regenerating system, were purchased from IPHASE (Suzhou, China). All reagents and solvents were of analytical or mass spectrometry grade.

### 2.2. LC-MS/MS Methods

An AB SCIEX Instruments ZenoTOF™ 7600 (Framingham, MA, USA) equipped with a Waters ACQUITY ultra performance liquid chromatograph (Milford, MA, USA) was used for sample analysis. The separation of analytes was carried out on a C18 column (4.6 × 250 mm, 5 μm; Agilent, Shanghai, China). A gradient elution was performed using (A) water and (B) 0.1% formic acid in acetonitrile at 0.2 mL/min on a column maintained at 30 °C. Gradient program: 0–8 min, 7–25% A; 8–20 min, 25–35% A; 20–21 min, 35–70% A; 21–24 min, 70–95% A; 24–26 min, 95–8% A; and 26–30 min, 8% A. The injection volume was 3 μL. Analysis was conducted using Zeno-TOF mass (Framingham, MA, USA) spectrometry with positive electrospray ionization. Source parameters included: sheath gas (N_2_), 50 psi; auxiliary gas (N_2_), 40 psi; nebulizing gas, 55 psi; heating gas, 55 psi; heater temperature, 550 °C; collision gas, 8 psi; capillary voltage, 5500 V; and declustering potential, 80 V. Full scan data (*m*/*z* 100–900) were acquired with a collision energy of 12 eV and an accumulation time of 0.15 s. For data-dependent acquisition, product ion scans used a collision energy of 35 eV, an accumulation time of 0.015 s, and a total cycle time of 0.804 s.

### 2.3. HPLC Methods

A Waters Alliance 2695 HPLV-DAD system (Milford, MA, USA), equipped with a Waters 2695 separations module, an adjustable temperature oven, and Empower 3 data processing software, was used for analysis. For maclekarpine E, we established an HPC-DAD method to determine its concentration. Chromatographic conditions: Column: XAqua C18 (5 μm, 2.1 × 150 mm). Mobile phase: A: 0.1% phosphoric acid in water; B: acetonitrile. Gradient elution program: 0–8 min, 8–20% A; 8–20 min, 20–30% A; 20–21 min, 30–95% A; 21–24 min, 95–95% A; 24–26 min, 95–8% A; 26–30 min, 8–8% A. Detection wavelength: 270 nm. Column temperature: 35 °C. Flow rate: 0.5 mL/min. Injection volume: 25 μL.

### 2.4. Stability in Artificial Gastric and Intestinal Fluids

Artificial gastric fluid (AGF, pH 1.2) and artificial intestinal fluid (AIF, pH 6.8) were purchased from Scientific Phygene (Fuzhou, China). A stock solution of maclekarpine E (3 mg/mL) was prepared in 5% DMSO. Working solutions were obtained by diluting 200 μL of the stock solution with 1800 μL of either AGF or AIF, and the mixtures were incubated at 37 °C. For the gastric stability assay [12], 100 μL aliquots were withdrawn at 15, 30, 45, 60, 75 and 90 min; for the intestinal stability assay, aliquots were taken at 0.5, 1, 2, 4 and 6 h. Each aliquot was immediately quenched with 900 μL of mobile phase, centrifuged at 10,000 rpm for 10 min at 4 °C, and the supernatant was collected for HPLC semi-quantitative analysis. All experiments were performed in triplicate.

### 2.5. Maclekarpine E Metabolism by CYP450

The cytochrome P450 (CYP) phenotyping assay was performed using five major recombinant human CYP isoforms (CYP1A2, CYP2C9, CYP2C19, CYP2D6, and CYP3A4). Each isoform (final concentration 0.5 μM) was separately incubated in 0.1 M Tris-HCl buffer (pH 7.4) containing an NADPH-regenerating system (1.3 mM NADP^+^, 3.3 mM glucose-6-phosphate, 0.4 U/mL glucose-6-phosphate dehydrogenase, and 3.3 mM magnesium chloride). Maclekarpine E was added to a final concentration of 0.6 μM to initiate the reaction. After incubation at 37 °C for 30 min, the reaction was terminated by adding an equal volume (500 μL) of ice-cold acetonitrile containing 1% trifluoroacetic acid. The mixture was vortexed thoroughly and centrifuged at 10,000× *g* for 10 min at 4 °C. The supernatant was collected and subjected to HPLC for Semi-quantitative Analysis of the residual parent compound. Negative control samples were prepared identically but without the NADPH-regenerating system. All incubations were performed in triplicate.

### 2.6. In Vivo Metabolic Study of Maclekarpine E in Rats

#### 2.6.1. Animal Treatment and Sample Collection

Male Sprague-Dawley rats (180–200 g, 4–5 weeks old) were provided by Hunan SJA Laboratory Animal Co., Ltd. (Changsha, China) and acclimatized for one week under standard laboratory conditions. The rats were randomly divided into four groups (*n* = 4 per group): Group I for plasma collection, Group II for urine and feces collection, and two blank control groups. Maclekarpine E was dissolved in 1% DMSO to a final concentration of 50 mg/mL and administered orally at a dose of 250 mg/kg once daily for five consecutive days. The dose of 250 mg/kg was selected based on preliminary tolerability studies (LD_50_ > 2500 mg/kg) and to ensure sufficient exposure for metabolite detection in biosamples. Blood samples were collected at 2 h and 6 h after the last administration. Urine and fecal samples were collected over three-time intervals (0–6 h, 6–12 h, and 12–24 h) post-dose [13]. Blank samples were collected prior to dosing. All animal experiments were approved by the Institutional Animal Care and Use Committee of Hunan Agricultural University (Approval No. HUNAULSK 20250310–03) and conducted in accordance with the guidelines for the Care and Use of Laboratory Animals.

#### 2.6.2. Sample Pretreatment

Sample preparation procedures were performed with reference to Liu et al. [14]. Plasma samples (250 μL) were mixed with 1 mL of acetonitrile (MeCN) to precipitate proteins, vortexed for 5 min, and centrifuged at 10,000 rpm for 10 min at 4 °C. The supernatant was transferred and evaporated to dryness under a gentle stream of nitrogen at 40 °C. The residue was reconstituted in 100 μL of MeCN, vortexed for 5 min, and centrifuged again. The final supernatant was collected for analysis. Urine samples (1 mL) were mixed with an equal volume of MeCN, vortexed for 2 min, and centrifuged under the same conditions. The supernatant was dried under nitrogen and reconstituted in 200 μL of MeCN, followed by centrifugation. The clear supernatant was collected. Fecal samples (approximately 0.5 g) were ultrasonically extracted with 10 mL of MeCN for 30 min. The extract was centrifuged at 10,000 rpm for 10 min, and the supernatant was dried under nitrogen. The residue was reconstituted in 200 μL of MeCN, centrifuged, and the supernatant was collected. All samples were filtered through a 0.22 μm membrane prior to UPLC-ZenoTOF analysis.

### 2.7. Molecular Docking Validation

To investigate whether biliary efflux transporters contribute to the pronounced fecal accumulation of maclekarpine E and its metabolites, molecular docking was performed against human ABCG2 (BCRP) and ABCC2 (MRP2) using the Schrödinger Maestro 12.8 suite. All docking calculations were carried out with Glide in standard precision (SP) mode and the OPLS4 force field.

The cryo-EM structures of ABCG2 (PDB ID: 6VXJ) and ABCC2 (PDB ID: 9C12) were retrieved from the RCSB Protein Data Bank [15]. Protein preparation was conducted with the Protein Preparation Wizard, including hydrogen addition, assignment of bond orders, removal of crystallographic water molecules, and restrained energy minimization. The co-crystallized ligands were removed prior to grid generation. For each transporter, a receptor grid (20 Å × 20 Å × 20 Å) was centered on the reported substrate-binding cavity.

Twenty fecal compounds (maclekarpine E and its 19 metabolites) were prepared as ligands using LigPrep; ionization states at pH 7.4 ± 1.0 were generated with Epik, and energy minimization was performed with OPLS4. Leukotriene C4 and SN38 were used as positive control substrates for ABCG2 and ABCC2, respectively [16,17]. All ligands were flexibly docked, and up to ten poses per ligand were retained. The best pose was selected based on the lowest Glide score (ΔG, kcal/mol). A ΔG value below –5.0 kcal/mol was considered indicative of favorable binding [18]. Interaction diagrams were analyzed to identify key residues involved in ligand–transporter recognition.

### 2.8. Network Pharmacology Analysis

To explore the potential mechanisms of maclekarpine E metabolites against ulcerative colitis (UC), a network pharmacology approach was employed. A total of 20 compounds, including maclekarpine E and its 19 metabolites identified in rat feces, were selected as candidate chemicals. Their 2D structures (SMILES format) were generated using InDraw version 7.1 and standardized with OpenBabel version 3.0.1. For metabolite target prediction, the SMILES strings of the 20 compounds were submitted to the SwissTargetPrediction platform (http://www.swisstargetprediction.ch, 2019 version) [19] with the organism set to Homo sapiens. All predicted targets with a probability > 0 were retained. To improve coverage, the same compounds were also submitted to the SEA Search Server (https://sea.bkslab.org, version 3.0) based on the Similarity Ensemble Approach [20], and all predicted human targets with *p* < 0.05 were retained. The results from both databases were merged, duplicates removed, and all protein identifiers were converted to official gene symbols using UniProt (https://www.uniprot.org) [21]. This merged set was defined as the potential metabolite targets. UC-associated targets were retrieved from the GeneCards database (https://www.genecards.org, version 5.22) [22] using the keyword “ulcerative colitis”. Only genes with a relevance score ≥ 1.0 were selected. After deduplication, the disease target set was obtained. The metabolite targets and disease targets were intersected using Venny 2.1.0 (https://bioinfogp.cnb.csic.es/tools/venny/ (accessed on 25 November 2025)). The overlapping genes were defined as the putative therapeutic targets of maclekarpine E metabolites against UC and were used for subsequent analyses.

These overlapping targets were imported into the STRING database (https://cn.string-db.org, version 12.0) [23] with the species limited to Homo sapiens and the minimum required interaction score set to 0.7 (high confidence). Disconnected nodes were removed, and the interaction data were exported and visualized in Cytoscape 3.10.0. Hub genes were identified using the cytoHubba plugin with the Maximal Clique Centrality (MCC) algorithm. The top 20 genes ranked by MCC were displayed as the hub subnetwork and selected as core hub genes for further enrichment analysis. Functional annotation of the 20 core hub genes was performed using the DAVID database (https://davidbioinformatics.nih.gov/, version 6.8) [24]. Gene Ontology (GO) enrichment analysis was conducted for Biological Process (BP), Cellular Component (CC), and Molecular Function (MF), and KEGG pathway enrichment analysis was performed against the KEGG Pathway database. The significance thresholds were set as gene count ≥ 2 and EASE Score < 0.05. A metabolite–target–disease interaction network was constructed in Cytoscape to visualize the relationships among the 20 metabolites, the overlapping targets, and UC. Topological parameters (degree, betweenness centrality) were calculated using the Network Analyzer tool. All enrichment results were visualized by https://www.bioinformatics.com.cn (last accessed on 12 February 2026), an online platform for data analysis and visualization [25].

### 2.9. Statistical Analysis

All data are presented as mean ± standard deviation (SD) of three independent experiments. Statistical analyses were performed using GraphPad Prism (version 9.0, GraphPad Software, San Diego, CA, USA). Normality of data distribution was assessed using the Shapiro–Wilk test. For comparisons between multiple groups, one-way analysis of variance (ANOVA) was conducted, followed by Dunnett’s post hoc test for multiple comparisons against the control group. Statistical significance was set at *p* < 0.05. For CYP phenotyping, substrate depletion in each enzyme group was compared to the NADPH-free negative control.

## 3. Results

### 3.1. Stability of Maclekarpine E in Artificial Gastric and Intestinal Fluids

**Figure 1:**
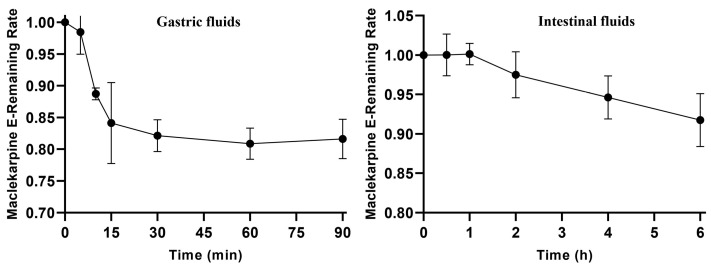
Stability of maclekarpine E in artificial gastric fluid (AGF, pH 1.2) and artificial intestinal fluid (AIF, pH 6.8) at 37 °C.

The stability of maclekarpine E was evaluated under simulated gastrointestinal conditions by incubation in AGF (pH 1.2) and AIF (pH 6.8) at 37 °C. The residual percentages of the parent compound at each time point are presented in Figure 1. In AGF, maclekarpine E underwent rapid degradation within the first 15 min, with approximately 15% of the compound lost. Thereafter, the residual level remained relatively stable, fluctuating between 78% and 85% throughout the remaining 90 min incubation period. These results indicate that maclekarpine E possesses moderate acid stability and that the initial degradation phase is followed by a plateau. In contrast, maclekarpine E was completely stable in AIF during the 90 min incubation, with 100% of the compound retained at all time points. Extended incubation up to 6 h further confirmed its excellent stability in simulated intestinal fluid, with a residual rate of 90%. Collectively, the favorable stability profiles in both gastric and intestinal environments support the oral applicability of maclekarpine E and provide a solid foundation for subsequent in vivo metabolism studies.

### 3.2. MS/MS Fragmentation Pattern of Maclekarpine E

**Figure 2:**
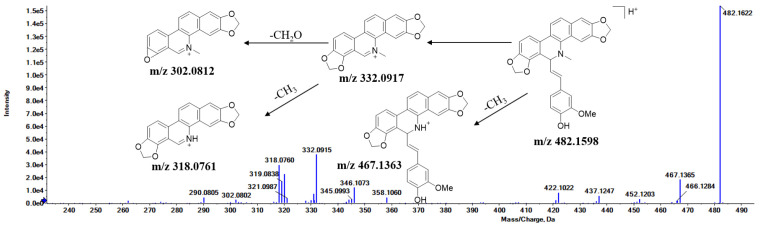
The mass spectrometric fragmentation pattern of maclekarpine E.

A detailed MS/MS fragmentation study of maclekarpine E was conducted to facilitate the metabolite identification. As shown in Figure 2, the protonated molecule [M + H]^+^ was observed at *m*/*z* 482.1622. The product ion at *m*/*z* 467.1363 originated from the direct loss of a methyl radical (·CH3), a fragmentation behavior characteristic of dihydrobenzophenanthridine alkaloids. Additional diagnostic fragments were observed at *m*/*z* 332.0917, 318.0761, and 202.0812, corresponding to the cleavage of the feruloyl moiety (loss of C9H10O2), further dissociation of the side chain, and the isoquinoline core, respectively. The fragmentation pattern of maclekarpine E closely resembles those reported for sanguinarine [14,26,27] and chelerythrine [28], providing a reliable basis for subsequent metabolite identification.

### 3.3. Identification of Maclekarpine E Metabolites in Rat Plasma, Urine and Feces by UPLC–ZenoTOF

Metabolite identification followed established confidence criteria (Metabolomics Standards Initiative) [29]: (i) exact mass error predominantly <5 ppm (with a maximum tolerance of 6 ppm) for molecular formula assignment; (ii) diagnostic fragment ions matching the parent compound’s fragmentation pattern or rational biotransformation pathways; (iii) retention time consistency with predicted polarity; and (iv) comparison with literature reports of structurally related alkaloids where available. A total of 19 metabolites (M1–M19) were unequivocally or tentatively identified in rat plasma, urine, and feces after oral administration of maclekarpine E by UPLC–ZenoTOF. Their retention times, accurate masses, elemental compositions, characteristic fragment ions, mass errors (<6 ppm), and biological distributions are summarized in Table 1. Representative extracted ion chromatograms and the MS/MS spectra of all metabolites are provided in the Figures S1–S23. The parent compound (M0, [M + H]^+^ at *m*/*z* 482.1622, C_29_H_23_NO_6_) was detected in plasma, urine, and feces. Based on accurate mass measurements and diagnostic fragmentation behavior, the biotransformation pathways of maclekarpine E were elucidated as ring-opening, demethylation, oxidation, and their combinations.

M1–M4 were eluted at 14.05, 14.17, 14.58, and 14.93 min, respectively. M1 and M2 displayed an identical [M + H]^+^ ion at *m*/*z* 484.1746 (C_29_H_25_NO_6_, Δ −3.1 ppm), corresponding to a 2 Da increase relative to M0, which indicates ring-opening of methylenedioxy of M0. Their MS/MS spectra all exhibited characteristic fragment ions at *m*/*z* 469.1506 ([M + H–CH_3_]^+^), 334.1068, and 320.0918, supporting the proposed structures. These four metabolites were predominantly found in feces and plasma, while M2 was also detectable in urine.

M5–M8 (retention times: 13.06, 13.35, 13.25, and 13.63 min) shared an identical [M + H]^+^ ion at *m*/*z* 470.1586 (C_29_H_23_NO_7_, Δ −3.3 to +5.8 ppm), 14 Da lower than M1–M4, suggesting demethylation. The product ions at *m*/*z* 455.1353 ([M + H–CH_3_]^+^), 334.1062, and 320.0911 indicated that ring open and demethylation occurred on the parent skeleton while the feruloyl side chain remained intact. These metabolites were detected mainly in feces and plasma.

M9 was eluted at 15.03 min and exhibited a protonated molecule at *m*/*z* 456.1447 (C_29_H_23_NO_7_, Δ +1.9 ppm), as well as a demethylation product. Its fragmentation produced ions at *m*/*z* 441.1202 ([M + H–CH_3_]^+^), 320.0917, and 306.0762, indicating a different demethylation site from M5–M8. M9 was exclusively detected in feces.

M10 (RT = 9.25 min) gave an [M + H]^+^ ion at *m*/*z* 444.1434 (C_26_H_21_NO_6_, Δ +2.1 ppm), representing a 38 Da decrease from M0. This mass shift is consistent with the loss of C_3_H_2_O and suggests a combination of ring-opening and multiple demethylations. Diagnostic fragments at *m*/*z* 429.1195, 308.0925, and 293.0684 support this assignment. M10 was found only in feces.

M11 (RT = 14.89 min) displayed [M + H]^+^ at *m*/*z* 468.1447 (C_28_H_21_NO_6_, Δ +1.5 ppm), 14 Da lower than M0, corresponding to demethylation. Its MS/MS spectrum showed ions at *m*/*z* 453.1200 ([M + H–CH_3_]^+^), 332.0914, and 318.0755, closely resembling the fragmentation of M0. M11 was exclusively present in feces.

M12 and M13 (RT = 10.22 and 10.35 min) shared an [M + H]^+^ ion at *m*/*z* 458.1604 (C_27_H_23_NO_6_, Δ +1.1 and +2.3 ppm), 24 Da lower than M0. The mass decrease indicates the loss of C_2_H_4_, rationalized as ring-opening accompanied by demethylation. Characteristic fragment ions at *m*/*z* 443.1344, 308.0917, and 293.0806 were observed. Both metabolites were detected only in feces.

M14 and M15 (RT = 13.74 and 14.13 min) exhibited [M + H]^+^ at *m*/*z* 498.1543 (C_29_H_23_NO_7_, Δ +1.5 and +5.6 ppm), another pair of mono-oxygenated isomers. Their MS/MS spectra displayed fragments at *m*/*z* 483.1306 ([M + H–NCH_3_]^+^), 348.0857, and 334.0696, suggesting hydroxylation at the benzophenanthridine core. These metabolites were found in both feces and plasma.

M16–M19 (RT = 12.14, 13.31, 14.73, and 15.03 min) shared an [M + H]^+^ ion at *m*/*z* 500.1707 (C_29_H_25_NO_6_, Δ −2.3 to +4.2 ppm), indicating that they are also oxidation products of M1–M4. Their fragmentation ions at *m*/*z* 485.1433 ([M + H]^+^) and 350.1005, these fragment ions suggest that the oxidation site is primarily located on the benzene ring of the benzophenanthridine moiety, rather than on that of the guaiacyl structural unit. These metabolites were exclusively detected in feces.

Notably, the majority of metabolites (19 out of 19) were detected in feces, whereas only 9 metabolites were found in plasma and 2 in urine (M0 and M2). This pronounced enrichment in feces suggests that biliary excretion may play a dominant role in the disposition of maclekarpine E and its metabolites. Furthermore, no phase II conjugates (glucuronides or sulfates) were identified in any of the biological matrices, consistent with the presence of a free hydroxyl group in the parent structure and potential steric hindrance that limits conjugation.

### 3.4. Metabolism of Maclekarpine E by Recombinant CYP Enzymes

To identify the CYP isoforms responsible for the oxidative metabolism of maclekarpine E, the compound was incubated with five major recombinant human CYP enzymes (CYP1A2, CYP2C9, CYP2C19, CYP2D6, and CYP3A4) in the presence of an NADPH-regenerating system. The metabolic activity was assessed by quantifying the residual amount of the parent compound using HPLC-UV, and the results are expressed as the percentage of substrate depletion relative to enzyme-free controls.

**Figure 3:**
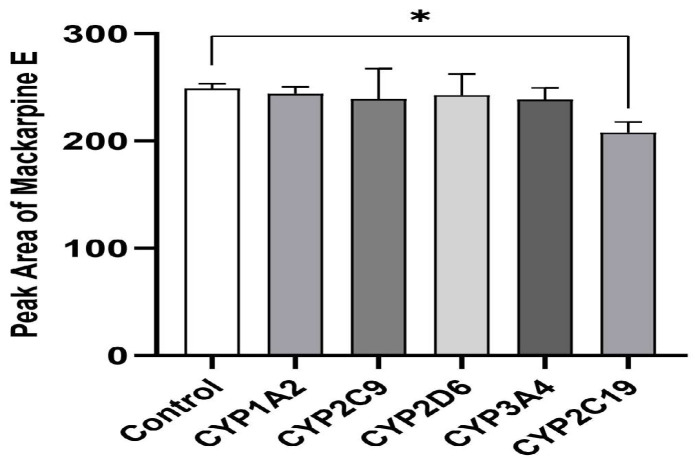
Depletion of maclekarpine E by five recombinant human CYP enzymes. * represents *p* < 0.05.

As shown in Figure 3, among the five tested CYP isoforms, only CYP2C19 exhibited statistically significant metabolic turnover of maclekarpine E compared to the negative control (*p* < 0.05), with a depletion rate of approximately 16.5%. The other four isoforms showed no significant substrate depletion under the same experimental conditions. These results demonstrate that CYP2C19 is the principal CYP isoform responsible for the oxidative biotransformation of maclekarpine E.

### 3.5. Proposed Metabolic Pathways of Maclekarpine E in Rats

**Figure 4:**
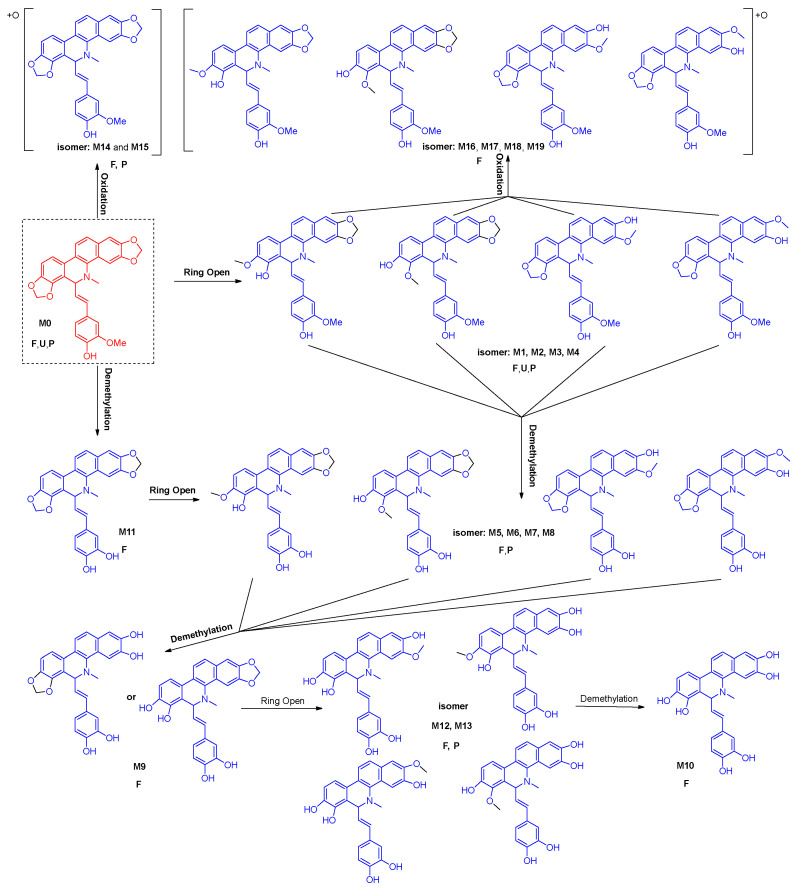
The proposed metabolic pathways of maclekarpine E in rats. Note: red represents parent compound, blue represents metabolite; F, feces; U, urine; P, plasma.

Based on the comprehensive profiling of metabolites in rat plasma, urine, and feces, the major biotransformation pathways of maclekarpine E are proposed in Figure 4. The parent compound (M0) underwent three primary types of metabolic modifications: ring-opening (reduction of the iminium bond), O-demethylation, and oxidation, either singly or in combination.

Ring-opening of the benzophenanthridine skeleton produced M1, M2, M3 and M4, along with their oxidation product M16–M19 and demethylation product M5–M8, which shared the same molecular formula but exhibited distinct chromatographic behaviors. M5–M8 can undergo further ring opening to form M12, M13, which continues demethylation to obtain M10. Also, the direct O-demethylation of M0 yielded M11. On the other hand, M0 can undergo direct oxidation to generate metabolites M14 and M15.

Notably, all identified metabolites were phase I products; no glucuronide or sulfate conjugates were detected in any biosample. The pronounced enrichment of metabolites in feces (19 out of 19) compared to plasma (9) and urine (2) indicates that biliary excretion is the dominant elimination route for maclekarpine E and its biotransformation products.

### 3.6. Molecular Docking Assessment of Potential Interactions with Biliary Efflux Transporters

To explore whether active biliary efflux may contribute to the pronounced fecal enrichment of maclekarpine E and its metabolites, molecular docking was performed against human ABCG2 (BCRP) and ABCC2 (MRP2). A total of 20 fecal compounds (the parent compound M0 and its 19 metabolites) were docked into the substrate-binding cavities of the respective transporters using the Glide SP protocol. Binding affinity was evaluated as the lowest Glide score (ΔG, kcal/mol), with a threshold of ΔG < −5.0 kcal/mol defined as indicative of favorable interaction. The docking protocol was validated using established positive controls. Leukotriene C4, a known ABCG2 substrate, yielded a binding energy of −7.989 kcal/mol, while SN38, a prototypical ABCC2 substrate, exhibited a Glide score of −11.094 kcal/mol, both well below the favorable binding threshold and confirming the reliability of the docking parameters.

**Figure 5:**
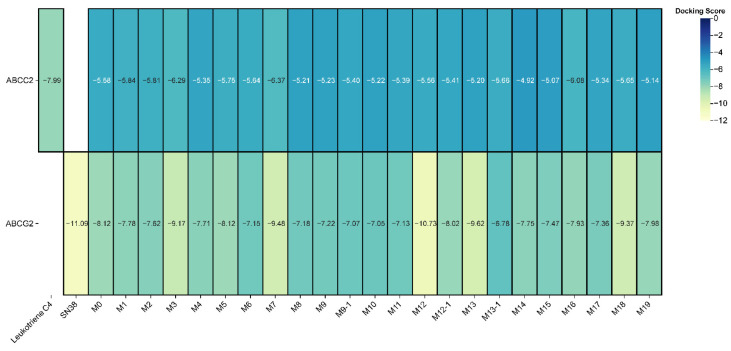
Molecular docking validation of biliary excretion. Heatmap of predicted binding free energies (Glide score, ΔG in kcal/mol) for 20 fecal compounds (maclekarpine E and its 19 metabolites; isomeric forms are denoted as −1) with human ABCG2 (PDB: 6VXJ) and ABCC2 (PDB: 9C12).

ABCG2 showed broad substrate acceptance: all 20 tested compounds exhibited binding energies below −5.0 kcal/mol (Figure 5). Notably, several metabolites displayed exceptionally strong affinities, including M12 (−10.729 kcal/mol), M13 (−9.618 kcal/mol), M7 (−9.479 kcal/mol), M18 (−9.369 kcal/mol), and M3 (−9.173 kcal/mol). These results indicate that biotransformations such as ring-opening and demethylation can enhance structural complementarity to the ABCG2 binding pocket. For ABCC2, 19 out of the 20 compounds met the favorable binding threshold (ΔG < −5.0 kcal/mol). The only exception was one of the two isomeric forms of M14, which exhibited a binding energy above −5.0 kcal/mol, indicating unfavorable interaction. All other compounds, as well as the remaining isomer of M14, displayed binding energies below the threshold. This highlights the stringent stereochemical requirements of ABCC2 substrate recognition, where even subtle structural differences between isomers can determine transporter affinity.

**Figure 6:**
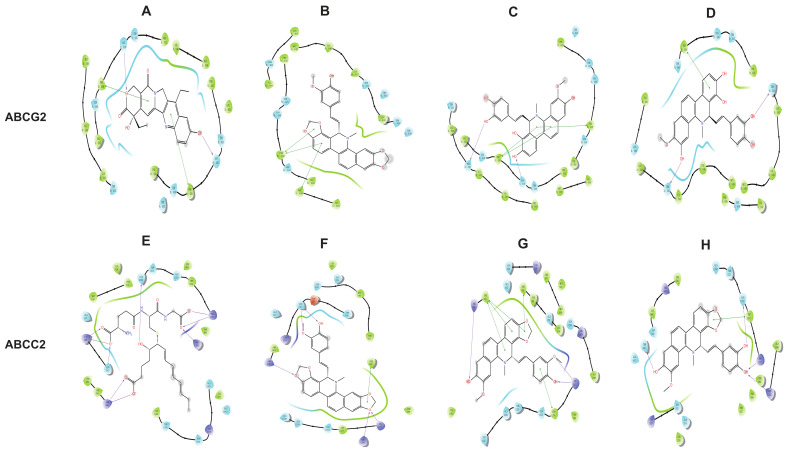
Two‑dimensional interaction diagrams of representative compounds with ABCG2 and ABCC2. (**A**–**D**) Binding poses of compounds with ABCG2 (PDB: 6VXJ). (**A**) Positive control SN38; (**B**) parent compound M0; (**C**) high‑affinity metabolite M12‑1; (**D**) metabolite M13‑1. (**E**–**H**) Binding poses of compounds with ABCC2 (PDB: 9C12). (**E**) Positive control leukotriene C4; (**F**) parent compound M0; (**G**) metabolite M3; (**H**) metabolite M7. Interactions are depicted as follows: purple arrows indicate hydrogen bonds (direction from donor to acceptor); green solid lines denote π–π stacking; red-blue gradient solid lines indicate salt bridges (red for acidic, blue for basic).

Representative binding modes were analyzed to visualize the structural basis of transporter selectivity (Figure 6). ABCC2 (MRP2): The positive control leukotriene C4 formed a combination of hydrogen bonds and salt bridges with Lys329, Arg590, and Arg1257, together with additional hydrogen bonds with Asn332 and Arg1205 (Figure 6A). The parent compound M0 engaged in π–π stacking with Phe382 and hydrogen bonds with Arg1205, Arg1257, Arg590, and Asn332 (Figure 6C). Metabolite M3 displayed enhanced aromatic interactions, forming π–π stacking with Phe591, Phe382, and Trp1254, complemented by hydrogen bonds with Arg590 and Arg1257 (Figure 6E). Metabolite M7 showed π–π stacking with Trp1254 and established hydrogen bonds with Thr336, Asn1253, Arg1257, and Arg1205. ABCG2 (BCRP): The positive control SN38 was anchored within the central hydrophobic cavity via π–π stacking with Phe439 from both chains (A and B) and hydrogen bonds with Asn436 from both chains (Figure 6B). The parent compound M0 exhibited three π–π stacking interactions with the conserved Phe439 residues of the two chains (Figure 6D). The highest-affinity metabolite M12-1 retained π–π stacking with Phe439 (both chains) and formed additional hydrogen bonds with Asn436 (chain A), Gln1250 (chain A), and Thr542 (chain B) (Figure 6G). Metabolite M13-1 displayed hydrogen bonds with Thr542 from both chains and π–π stacking with Phe439 (chain B).

Collectively, these docking results indicate that most maclekarpine E metabolites exhibit structural features compatible with recognition by ABCG2 and ABCC2. These findings support the hypothesis that active hepatobiliary efflux may contribute to the observed fecal accumulation, although functional transport assays are required for definitive confirmation.

### 3.7. Network Pharmacology Analysis of Fecal Metabolites

**Figure 7:**
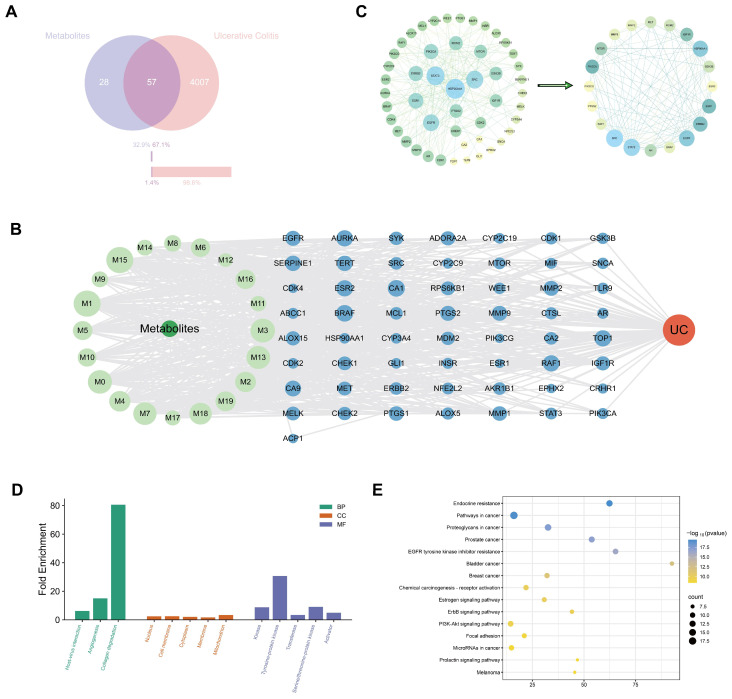
Network pharmacology analysis of maclekarpine E metabolites against UC. (**A**) Venn diagram of metabolite targets and UC disease targets. (**B**) Metabolite–target–disease network. Grass green represents metabolites, light green represents metabolites that meet screening criteria, blue represents targets, and red represents disease. (**C**) PPI network of overlapping targets and top 20 hub genes identified by MCC algorithm. (**D**) GO enrichment analysis of top 20 hub genes. (**E**) KEGG pathway enrichment analysis of top 20 hub genes (top 15 pathways).

To systematically explore the potential mechanisms by which maclekarpine E and its metabolites exert anti-ulcerative colitis effects, a network pharmacology approach was employed. The analysis workflow is illustrated in Figure 7, including compound-target prediction, protein–protein interaction (PPI) network construction, hub gene identification, and functional enrichment analysis.

A total of 20 compounds (maclekarpine E and its 19 metabolites identified in rat feces) were subjected to target prediction using public databases. After merging and removing duplicates, 85 non-redundant putative targets were obtained for these fecal metabolites (Figure 7A, left circle). Meanwhile, 4064 UC-associated targets were retrieved from disease gene databases (Figure 7A, right circle). The intersection of the two sets yielded 57 common targets, which were considered as the potential therapeutic targets of maclekarpine E metabolites against UC. To visualize the multi-component and multi-target relationships, a metabolite–target–disease network was constructed (Figure 7B). The network consisted of 20 metabolite nodes, 57 target nodes, and UC disease node, with edges representing interactions between metabolites and their corresponding targets. This network intuitively illustrates that maclekarpine E metabolites may act on UC through an integrative mode of multiple active compounds and multiple targets. The 57 overlapping targets were imported into the STRING database (confidence score > 0.7) to construct a PPI network. Isolated nodes were removed, resulting in a network containing 49 nodes and 198 edges (Figure 7C, left panel). The network was visualized using Cytoscape 3.10.0, and topological analysis was performed with the cytoHubba plugin. The top 20 hub genes ranked by the Maximal Clique Centrality (MCC) algorithm are highlighted in the core sub-network (Figure 7C, right panel). To further focus on the most central regulators, the top 10 hub genes (e.g., EGFR, STAT3, MAPK1, SRC, PIK3CA, etc.) were selected as core hub targets for subsequent enrichment analysis. To investigate the functional implications of the identified hub genes, the top 20 hub genes ranked by MCC algorithm were subjected to GO and KEGG enrichment analysis using DAVID with the threshold of count ≥ 2 and EASE Score < 0.05. The GO enrichment analysis revealed that the hub genes were significantly enriched in 3 Biological Process (BP) terms, 5 Cellular Component (CC) terms, and 5 Molecular Function (MF) terms (Figure 7D). GO analysis revealed that the hub genes were significantly enriched in three biological process terms, namely angiogenesis, collagen degradation, and host–virus interaction. In the cellular component category, the hub genes were predominantly localized to the nucleus, cytoplasm, cell membrane, membrane, and mitochondrion. For molecular function, significant enrichment was observed in kinase activity, protein tyrosine kinase activity, transferase activity, serine/threonine-protein kinase activity, and activator activity. KEGG pathway enrichment analysis identified a total of 96 significantly enriched pathways (adjusted *p* < 0.05). The top 15 pathways ranked by significance are shown in Figure 7E, including endocrine resistance, pathways in cancer, proteoglycans in cancer, and the PI3K-Akt signaling pathway, ErbB signaling pathway, and focal adhesion, among others.

## 4. Discussion

Maclekarpine E is a minor dihydrobenzophenanthridine alkaloid derived from *Macleaya* species, plants traditionally restricted to external use [30,31]. Our recent unpublished work first demonstrated its oral anti-colitic efficacy in mice, raising the critical question of whether the parent compound itself or its metabolites are responsible for this therapeutic effect. The present study provides the first comprehensive investigation into the in vivo metabolic fate of maclekarpine E, integrating metabolite profiling, CYP phenotyping, molecular docking validation, and network pharmacology. Our results delineate a distinctive disposition pattern characterized by extensive phase I metabolism, predominant biliary excretion, and a feces-enriched metabolite pool with predicted anti-inflammatory activities.

Maclekarpine E exhibited moderate acid stability in simulated gastric fluid, with approximately 15% degradation occurring within the first 15 min, followed by a plateau at 78–85% throughout the remaining 90 min incubation. The initial rapid degradation suggests that the compound undergoes partial chemical transformation under strongly acidic conditions. In contrast, maclekarpine E was highly stable in simulated intestinal fluid, retaining 100% of the parent compound during 90 min and 90% after extended 6 h incubation. This differential stability profile—moderate gastric loss followed by excellent intestinal persistence—is favorable for an orally administered agent targeting colonic diseases: it ensures that a substantial fraction of the dose reaches the lower gastrointestinal tract while avoiding excessive pre-absorptive degradation [32,33]. The partial gastric loss, while measurable, does not compromise the overall oral applicability of maclekarpine E and provides a solid foundation for its in vivo metabolic disposition.

A total of 19 phase I metabolites were unequivocally or tentatively identified in rats after oral administration, with a remarkable enrichment in feces (19/19) compared to plasma (9) and urine (2). This disproportionate distribution strongly suggests that biliary excretion, rather than passive diffusion or renal elimination, constitutes the dominant clearance route for maclekarpine E and its biotransformation products [34,35]. The primary biotransformation pathways identified—ring-opening of the methylenedioxy group, O-demethylation, and oxidation—are consistent with those reported for structurally related benzophenanthridine alkaloids such as sanguinarine and chelerythrine [14,28]. Notably, several isomeric metabolite pairs (e.g., M1–M4, M5–M8, M16–M19) were detected, reflecting the multiplicity of modification sites on the benzophenanthridine skeleton and the feruloyl side chain. The exclusive detection of M9, M10, M11, M12, M13, and M16–M19 in feces further implies that certain biotransformation products are either generated within the intestinal lumen (e.g., by gut microbiota) [36,37] or efficiently cleared via hepatobiliary efflux without re-entering the systemic circulation. Future studies using gnotobiotic animals or fecal microbiota incubation assays are warranted to delineate the relative contributions of host hepatic metabolism versus microbial metabolism to the observed fecal metabolite profile. To mechanistically validate the biliary excretion hypothesis, we performed molecular docking of all 20 fecal compounds against human ABCG2 (BCRP) and ABCC2 (MRP2), the two major apical efflux transporters mediating xenobiotic excretion into bile. The docking protocol was rigorously validated using established positive controls: leukotriene C4 for ABCG2 (ΔG = −7.989 kcal/mol) and SN38 for ABCC2 (ΔG = −11.094 kcal/mol), both of which closely reproduced their co-crystallized binding conformations [16,17]. Strikingly, all 20 compounds exhibited favorable binding affinities (ΔG < −5.0 kcal/mol) toward ABCG2, while 19 out of 20 compounds met the threshold for ABCC2. The sole exception was one of the two isomeric forms of M14, which displayed a binding energy above −5.0 kcal/mol. This finding highlights the stringent stereochemical requirements of ABCC2 substrate recognition, where even subtle differences in the spatial orientation of a hydroxyl group can determine transporter affinity [17,38]. The superior binding affinities of several metabolites—particularly M12 (−10.729 kcal/mol), M13 (−9.618 kcal/mol), and M7 (−9.479 kcal/mol) toward ABCG2—suggest that biotransformations such as ring-opening and demethylation may enhance structural complementarity to the transporter binding pocket. Detailed analysis of the interaction diagrams revealed that both the parent compound and its metabolites occupy the canonical substrate-binding cavities, forming abundant π–π stacking contacts with conserved phenylalanine residues (Phe382/Phe591/Trp1254 in ABCC2; Phe439 in ABCG2) and hydrogen bonds with polar/charged residues (e.g., Asn436, Arg590, Arg1257). These computational results suggest that ABCG2 and ABCC2 may play a role in the biliary excretion of maclekarpine E and its metabolites, offering a plausible explanation for their fecal enrichment. However, docking scores alone cannot establish functional substrate status, as they represent static affinity-based approximations rather than dynamic transport processes. Definitive validation requires quantitative uptake/efflux assays using transporter-overexpressing cell lines or bile duct-cannulated animal models.

The presence of the parent compound M0 in plasma, urine, and feces warrants further comment. Plasma detection of M0 confirms successful intestinal absorption, consistent with its favorable stability in simulated intestinal fluid (90% retention at 6 h). Urinary excretion of M0 (and M2) indicates that renal elimination operates as a minor, auxiliary clearance pathway for compounds that escape hepatic sequestration. The fecal detection of M0 can be attributed to two complementary sources: (i) unabsorbed fraction passing directly through the gastrointestinal tract, and (ii) absorbed fraction undergoing hepatobiliary efflux via ABCG2, as supported by its favorable docking score with this transporter (−7.13 kcal/mol). This multi-route disposition is typical of moderately lipophilic, passively permeable compounds [39] and does not contradict the bile-centric clearance model established for the more polar, bulky metabolites [40].

Notably, no glucuronide or sulfate conjugates were identified in any biosample. This absence may be attributed to several non-mutually exclusive possibilities. First, phase II metabolites, if formed, might be present at levels below the detection limit of the current analytical platform—a common challenge in metabolite profiling owing to their high polarity, poor ionization efficiency, and low abundance in complex biological matrices [41]. Second, from a structural perspective, the parent compound already possesses a free hydroxyl group, and additional hydroxyl groups introduced via oxidation may confer sufficient aqueous solubility, potentially reducing the driving force for further conjugation. Moreover, the bulky, fused benzophenanthridine skeleton may sterically hinder access to the active sites of UDP-glucuronosyltransferases (UGTs) and sulfotransferases (SULTs) [42]. Third, and mechanistically intriguing, the hydroxylated metabolites themselves may act as inhibitors of the conjugating enzymes. Although direct experimental evidence for this mechanism in the context of benzophenanthridine alkaloids is currently lacking, the structural and metabolic parallels with OH-PCBs and flavonoids provide a strong rationale for this hypothesis, which warrants dedicated investigation in future studies [43]. These OH-PCBs, which share structural features with the hydroxylated metabolites of maclekarpine E (e.g., M5–M8, M9, M14–M15)—namely, a polycyclic aromatic core bearing one or more phenolic hydroxyl groups—bind to the substrate-binding site of SULTs and competitively or noncompetitively inhibit the sulfation of both xenobiotics and endogenous substrates [44,45]. It is also possible that the absence of detectable phase II metabolites results from limited activity of conjugating enzymes toward these bulky, polycyclic structures, or from analytical limitations such as poor ionization or low abundance. Another speculative but mechanistically interesting possibility is that hydroxylated metabolites may inhibit sulfotransferases (SULTs) or UDP-glucuronosyltransferases (UGTs), as has been observed for hydroxylated polychlorinated biphenyls and flavonoids [46,47]. However, this hypothesis remains untested and requires dedicated investigation.

CYP phenotyping using recombinant human enzymes revealed that CYP2C19 significantly metabolized maclekarpine E, with a depletion rate of approximately 16.5% (*p* < 0.05), while the other tested isoforms showed minimal activity. This narrow isoform selectivity is consistent with the neutral, lipophilic nature of maclekarpine E. From a translational perspective, the involvement of CYP2C19 suggests that human metabolism of maclekarpine E may exhibit inter-individual variability and potential drug–drug interactions [48]. However, it is critical to note that these phenotyping experiments were conducted with human enzymes, whereas the in vivo metabolic profile was obtained from rats. While these phenotyping experiments were conducted with human enzymes, the in vivo metabolic profile was obtained from rats—a species that lacks a true ortholog of human CYP2C19. However, rat CYP2C enzymes, particularly the male-dominant Cyp2c11, exhibit partial functional overlap with human CYP2C19, including shared substrate specificities (e.g., omeprazole metabolism) [49,50]. This functional similarity supports the relevance of the rat model for identifying potential metabolic pathways, despite recognized limitations including sex-dependent expression patterns and differences in catalytic efficiency [51,52]. Therefore, while the observed involvement of CYP2C19 in human enzyme preparations and the functional similarities with rat Cyp2c11 support the relevance of our rat model for identifying potential metabolic pathways, direct quantitative extrapolation to humans must be undertaken with caution.

Network pharmacology was employed to generate hypotheses regarding the potential targets and pathways of the 20 fecal compounds. A total of 85 non-redundant targets were retrieved, intersecting with 57 UC-associated genes. Topological analysis of the PPI network identified 20 hub genes (e.g., EGFR, SRC, STAT3, MAPK1, PIK3CA, RAF1, BRAF) that occupy central positions in the interactome, implying their critical roles in mediating the pharmacological effects. GO enrichment of these hub genes pointed to angiogenesis, collagen degradation, and host–virus interaction as the most significantly overrepresented biological processes. Angiogenesis is a well-recognized pathological component of UC, where excessive and aberrant microvascular remodeling perpetuates chronic inflammation, impairs mucosal healing, and contributes to dysplasia risk [53,54]. Collagen degradation reflects extracellular matrix (ECM) turnover mediated by matrix metalloproteinases (MMPs); excessive ECM remodeling is intimately linked to epithelial barrier disruption, crypt distortion, and ulcer formation in active UC [55,56]. The “host–virus interaction” term, although seemingly distant, often encompasses genes involved in antiviral immunity and type I interferon signaling—pathways increasingly appreciated as modulators of intestinal inflammation and gut microbial homeostasis [32,57]. At the molecular function level, the hub genes were overwhelmingly enriched in kinase activities, particularly protein tyrosine kinase and serine/threonine-protein kinase activities. This kinase-centric signature underscores the centrality of phosphorylation-driven signaling cascades in UC pathogenesis and aligns with the successful clinical application of kinase inhibitors (e.g., tofacitinib, a JAK inhibitor) for UC treatment [58]. KEGG pathway enrichment further converged on the PI3K-Akt, ErbB, Ras, and Rap1 signaling pathways, as well as focal adhesion and proteoglycans in cancer. While these pathways are classically linked to oncogenesis, they are equally pivotal in regulating inflammation, cell proliferation, apoptosis, and barrier integrity in the intestinal epithelium [59,60,61,62]. The PI3K-Akt and MAPK axes, in particular, are central to the action of UC therapies and are frequently dysregulated in inflamed colonic mucosa [63,64]. The enrichment of “pathways in cancer” and tissue-specific cancer terms (breast, prostate, bladder) reflects the pleiotropic nature of the hub genes rather than a genuine oncogenic risk; many of these genes (e.g., EGFR, SRC, PIK3CA, BRAF) are signal transduction hubs that participate in diverse physiological and pathological processes, including inflammation and tissue repair [65,66]. Collectively, the network pharmacology analysis suggests that maclekarpine E metabolites may act through multi-target and multi-pathway mechanisms, with the PI3K-Akt and MAPK signaling cascades emerging as central nodes in this predictive model. These findings should be considered hypothesis-generating and require experimental validation.

Several limitations of this study should be acknowledged. First, although molecular docking provided compelling evidence that maclekarpine E and its metabolites are potential substrates of ABCG2 and ABCC2, these in silico predictions inherently represent static, affinity-based approximations and cannot fully recapitulate the dynamic transport processes in living systems. Definitive validation requires quantitative uptake/efflux assays using transporter-overexpressing cell lines (e.g., MDCK-BCRP/MRP2 monolayers) or bile duct-cannulated animal models. However, such direct evaluations are considerably hampered by the formidable difficulty in obtaining authentic standards of these phase I metabolites. Their low endogenous abundance in complex biological matrices, coupled with the structural complexity and isomerism of species such as M14 and M16–M19, renders large-scale isolation or chemical synthesis extremely challenging at present. In this context, molecular docking serves as a pragmatic and efficient surrogate approach to generate mechanistically testable hypotheses regarding biliary excretion. Future studies should prioritize the synthesis or biosynthesis of representative high-affinity metabolites (e.g., M12, M13, M7) to enable definitive transporter assays and pharmacokinetic evaluations. Second, the exact chemical structures of several isomeric metabolites—particularly M16–M19—could not be unambiguously assigned by MS/MS alone; NMR analysis or chemical synthesis is required for definitive structural confirmation. Third, while M9 was exclusively detected in feces and may implicate gut microbiota in its metabolism, direct evidence (e.g., fecal microbiota incubation studies or gnotobiotic animal models) is currently lacking. Fourth, the network pharmacology findings are inherently hypothesis-generating and await experimental verification through orthogonal techniques such as surface plasmon resonance, cellular thermal shift assays, or gene silencing in intestinal epithelial cells. Fifth, the present study focused on the metabolite profile in healthy rats; the metabolic disposition of maclekarpine E under colitic conditions—where inflammation-driven downregulation of CYPs and transporters is well documented [36]—remains to be investigated. Finally, the individual contribution of each metabolite, particularly the feces-specific, high-affinity ABCG2 substrates M12 and M13, to the overall anti-colitic efficacy observed in our murine study warrants dedicated in vivo dissection using metabolomics-guided pharmacodynamic approaches. Addressing these limitations in future work will not only solidify the mechanistic understanding of maclekarpine E disposition but also accelerate its development as a gut-targeted anti-inflammatory lead compound.

## 5. Conclusions

In summary, this work provides the first complete map of maclekarpine E metabolism in rats. The compound undergoes ring-opening, demethylation, and oxidation to yield 19 phase I metabolites, which are almost exclusively excreted into feces via ABCG2- and ABCC2-mediated biliary efflux. Molecular docking validated that all 20 fecal compounds are potential ABCG2 substrates, while ABCC2 exhibits stricter stereochemical selectivity. CYP2C19 is identified as the sole enzyme responsible for its oxidative biotransformation, a selectivity consistent with the neutral, lipophilic nature of the substrate. Network pharmacology predicts that the fecal metabolite pool may act as a multi-component, multi-target modulator of UC-relevant pathways, particularly the PI3K-Akt and MAPK signaling axes, with hub genes centered on kinases and ECM remodeling. These findings position maclekarpine E as a potential metabolically activated, gut-targeted anti-colitic lead, with its fecal metabolites predicted to target UC-relevant pathways. However, direct experimental validation of anti-inflammatory activity for individual metabolites is required to confirm this hypothesis.

## Figures and Tables

**Table 1:** Identification of maclekarpine E metabolites in rat plasma, urine and feces by UPLC-ZenoTOF-MS/MS.

Met No	T/min	Elemental Composition	Metabolic Pathway	Ionization	MS Fragment	MS/Error	Origin
M0	15.73	C_29_H_23_NO_6_	Parent	[M + H]^+^	332, 302;318;467	−3	P, F, U
M1	14.05	C_29_H_25_NO_6_	Ring open	[M + H]^+^	469.1506; 334.1068; 320.0918	−3.1	F, P
M2	14.17	C_29_H_25_NO_6_	Ring open	[M + H]^+^	469.1506; 334.1068; 320.0918	3.1	F, U, P
M3	14.58	C_29_H_25_NO_6_	Ring open	[M + H]^+^	469.1506; 334.1068; 320.0918	−2.9	F, P
M4	14.93	C_29_H_25_NO_6_	Ring open	[M + H]^+^	469.1506; 334.1068; 320.0918	1.7	F, P
M5	13.06	C_29_H_23_NO_7_	Demethylation	[M + H]^+^	455.1353; 334.1062; 320.0911	2.9	F, P
M6	13.35	C_29_H_23_NO_7_	Demethylation	[M + H]^+^	455.1353; 334.1062; 320.0911	1.2	F
M7	13.25	C_29_H_23_NO_7_	Demethylation	[M + H]^+^	455.1353; 334.1062; 320.0911	−3.3	F
M8	13.63	C_29_H_23_NO_7_	Demethylation	[M + H]^+^	455.1353; 334.1062; 320.0911	5.8	F, P
M9	15.03	C_29_H_23_NO_7_	Demethylation	[M + H]^+^	441.1202; 320.0917; 306.0762	1.9	F
M10	9.25	C_26_H_21_NO_6_	Ring Open and Demethylation	[M + H]+	429.1195; 308.0925; 293.0684	2.1	F
M11	14.89	C_28_H_21_NO_6_	Demethylation	[M + H]+	453.1200; 332.0914; 318.0755	1.5	F
M12	10.22	C_27_H_23_NO_6_	Ring Open and Demethylation	[M + H]^+^	443.1344; 308.0917; 293.0806	1.1	F
M13	10.35	C_27_H_23_NO_6_	Ring Open and Demethylation	[M + H]^+^	443.1344; 308.0917; 293.0806	2.3	F
M14	13.74	C_29_H_23_NO_7_	Oxidation	[M + H]^+^	467.1107; 348.0857; 334.0696	1.5	F, P
M15	14.13	C_29_H_23_NO_7_	Oxidation	[M + H]^+^	467.1107; 348.0857; 334.0696	5.6	F, P
M16	12.14	C_29_H_25_NO_6_	Ring Open and Oxidation	[M + H]^+^	485.1433; 350.1005	1.3	F
M17	13.31	C_29_H_25_NO_6_	Ring Open and Oxidation	[M + H]^+^	485.1433; 350.1005	4.2	F
M18	14.73	C_29_H_25_NO_6_	Ring Open and Oxidation	[M + H]^+^	485.1433; 350.1005	2.1	F
M19	15.03	C_29_H_25_NO_6_	Ring Open and Oxidation	[M + H]^+^	485.1433; 350.1005	−2.3	F

Notes: F, feces; U, urine; P, plasma.

## Data Availability

The original contributions presented in this study are included in the article/Supplementary Materials. Further inquiries can be directed to the corresponding authors.

## Supplementary Materials

The following supporting information can be downloaded at: https://www.mdpi.com/article/10.3390/cimb48030335/s1.
