# Supplementary material for: Biotransformation of Maclekarpine E in Rats: CYP2C19-Mediated Metabolism, Fecal Enrichment, and Network Pharmacology-Based Anti-Ulcerative Colitis Prediction
Source: Curr Issues Mol Biol. 2026 Mar 23;48(3):335. doi: 10.3390/cimb48030335 (PMC13024927; doi:10.3390/cimb48030335)
Supplement: Supplementary file 1 [file cimb-48-00335-s001.zip › Supplementary Materials(1).pdf]

## 1. The spectrum of EIC and TIC in plasma

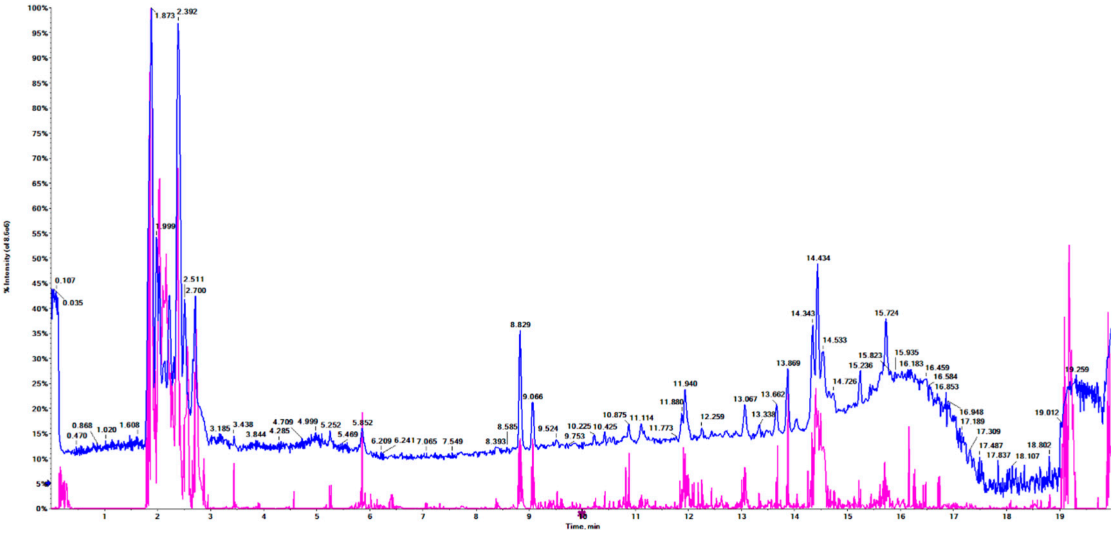


Fig.S1 TIC spectrum in plasma

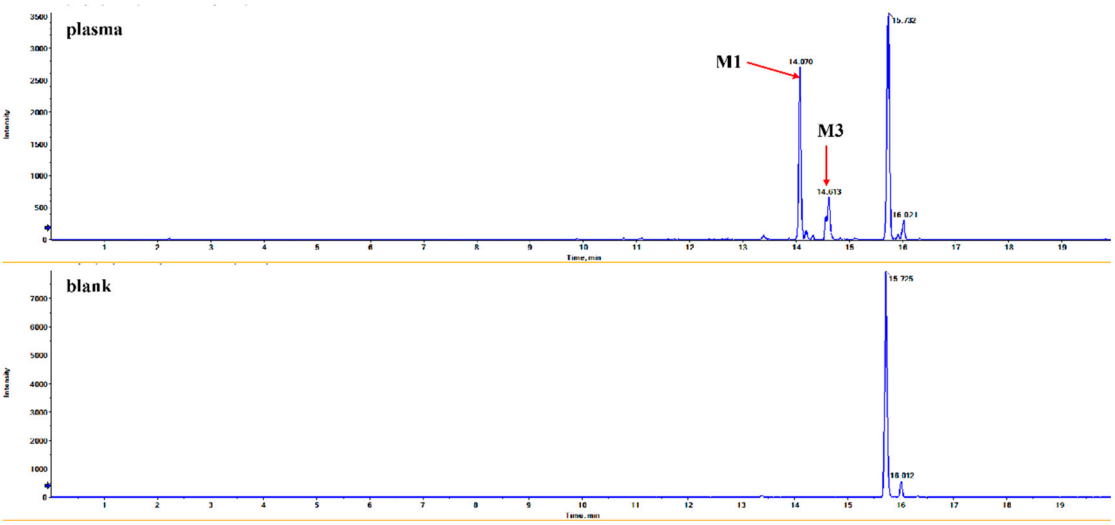


Fig.S2 EIC spectra of metabolites M1 and M3

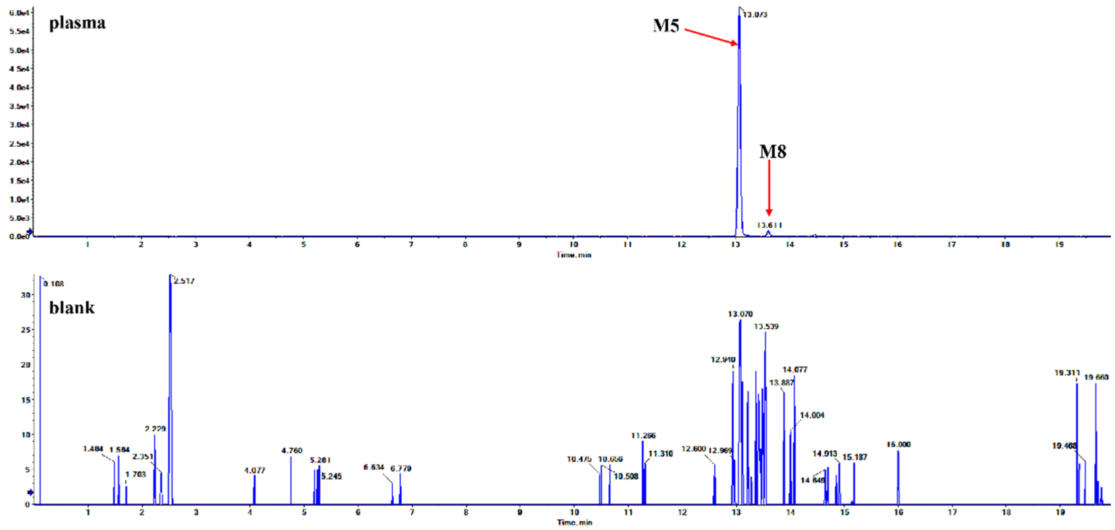


Fig.S3 EIC spectra of metabolites M5 and M8

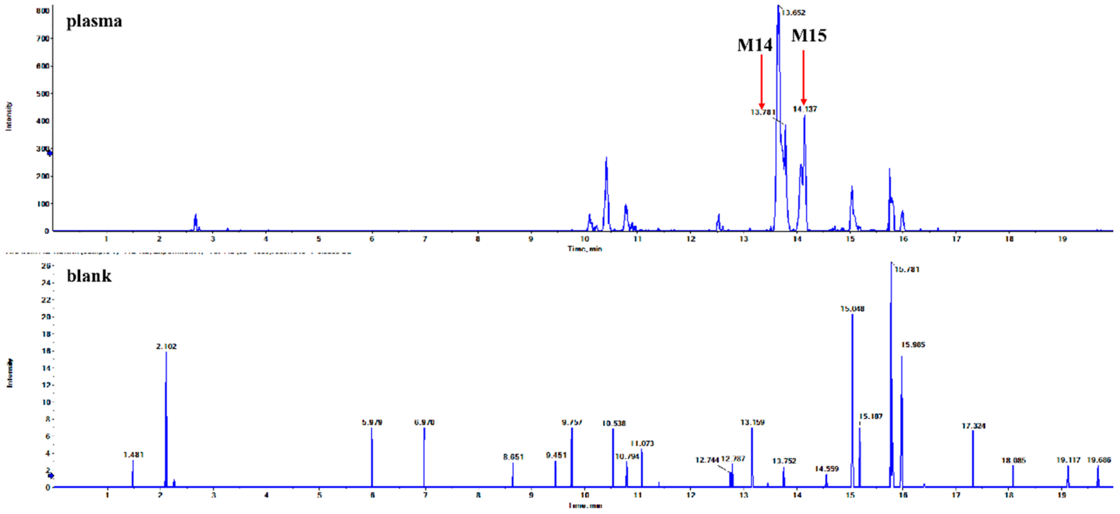


Fig.S4 EIC spectra of metabolites M14 and M15

## 2. The spectrum of EIC and TIC in urine

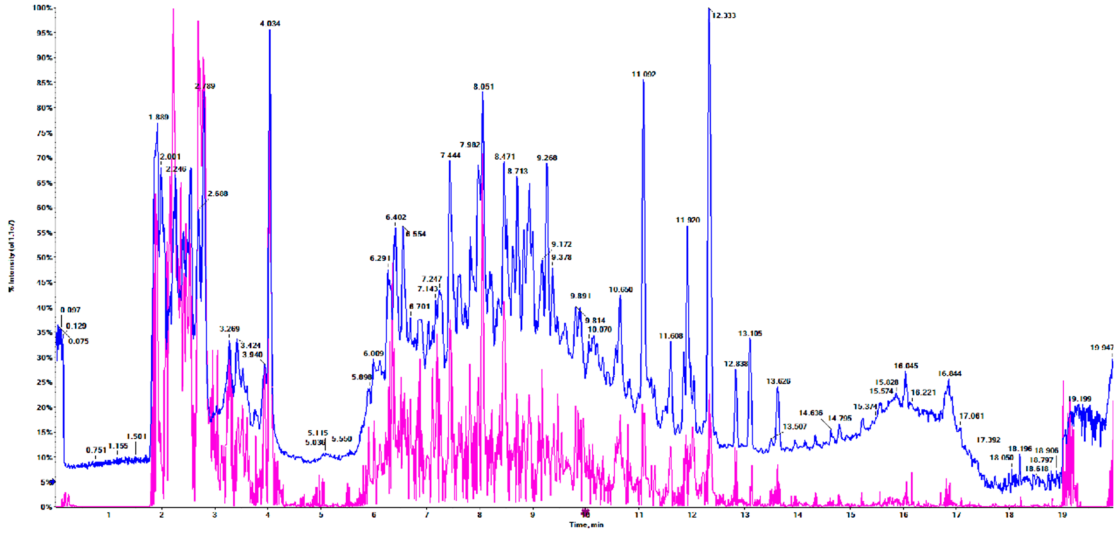


Fig.S5 TIC spectrum in urine

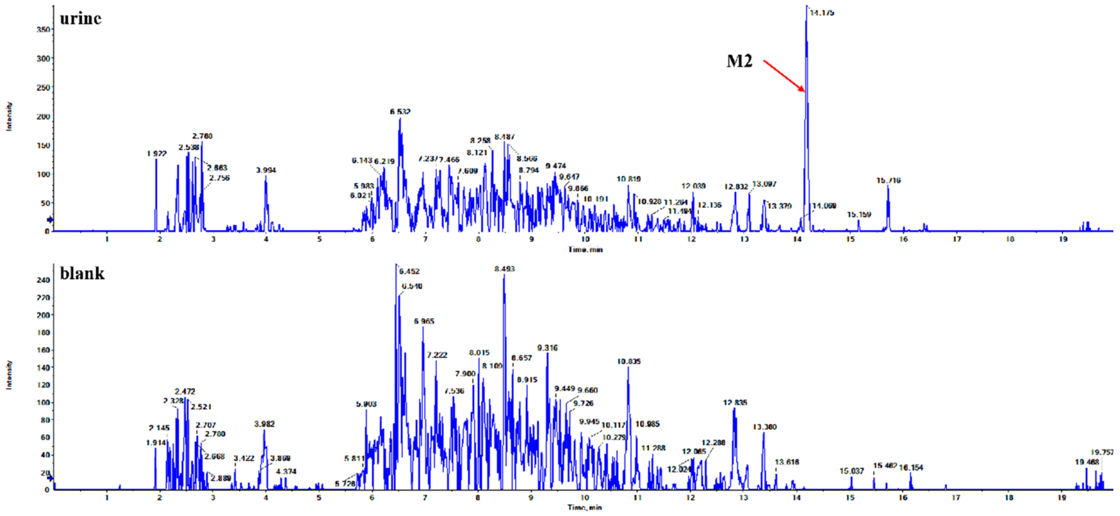


Fig.S6 EIC spectra of metabolites M12 in urine

## 3. The spectrum of EIC and TIC in feces

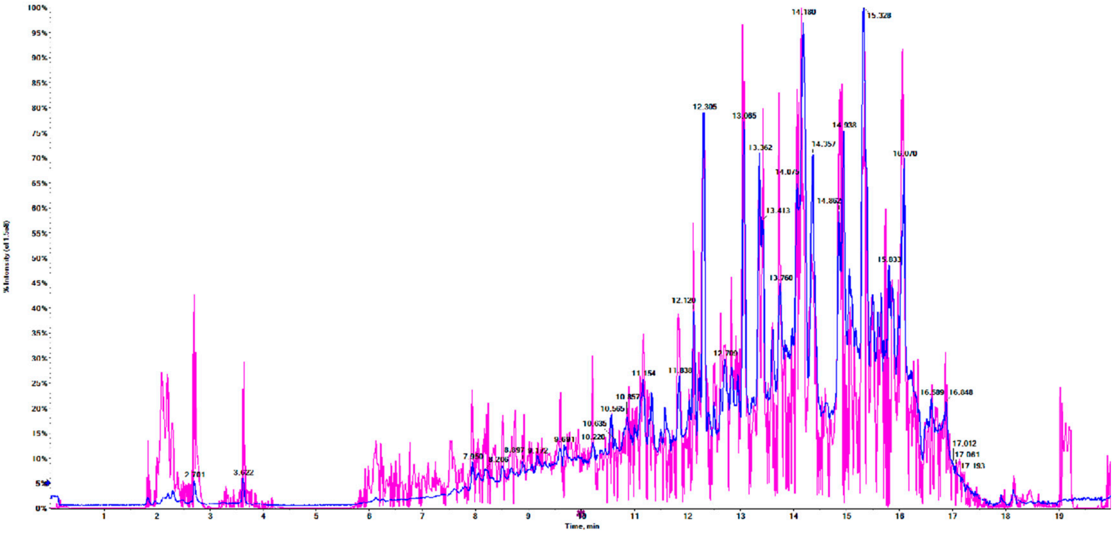

Fig.S7 TIC spectrum in feces

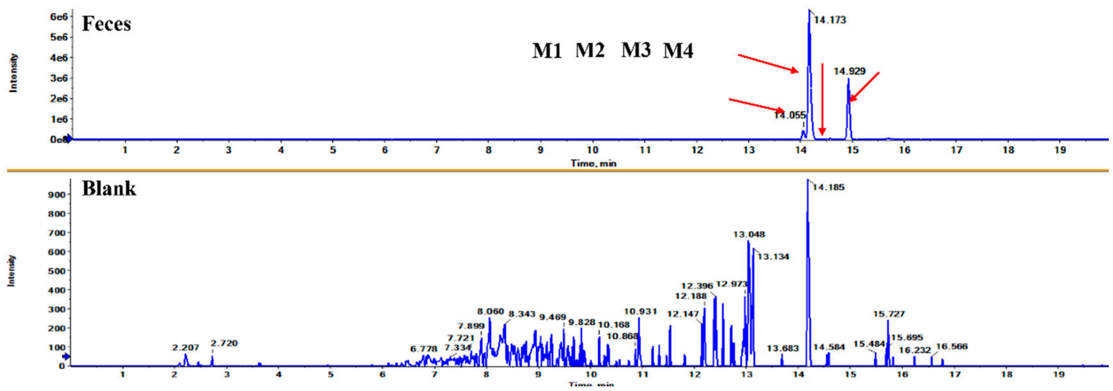


Fig.S8 EIC spectra of metabolites M1–M4 in feces

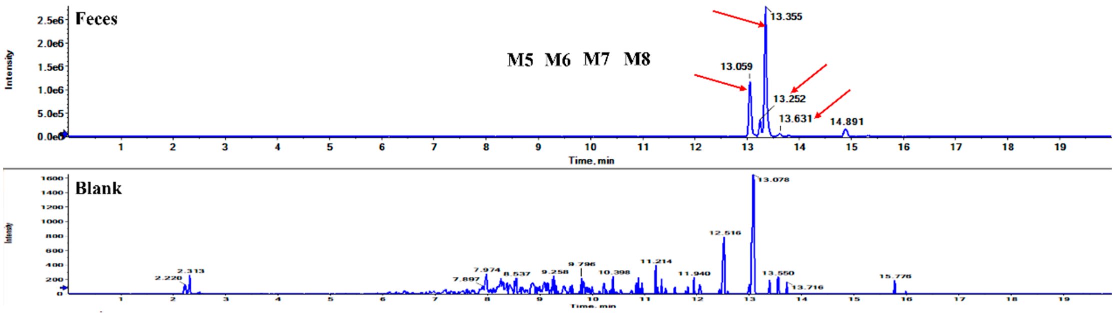


Fig.S9 EIC spectra of metabolites M5–M8 in feces

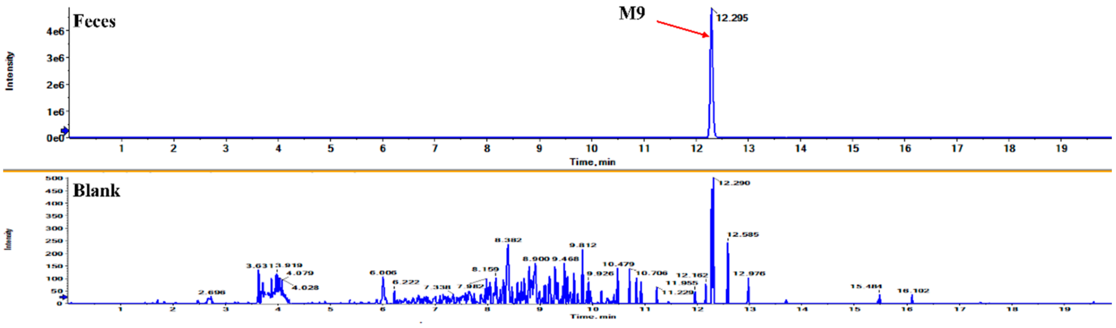

Fig.S10 EIC spectra of metabolites M9 in feces

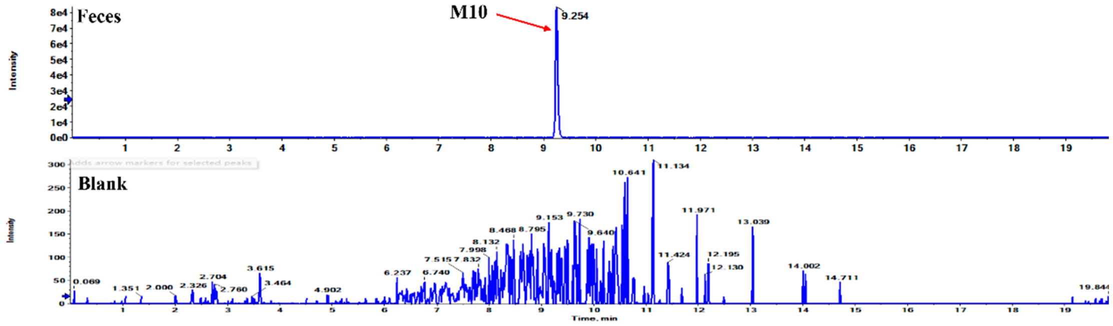


Fig.S11 EIC spectra of metabolites M10 in feces

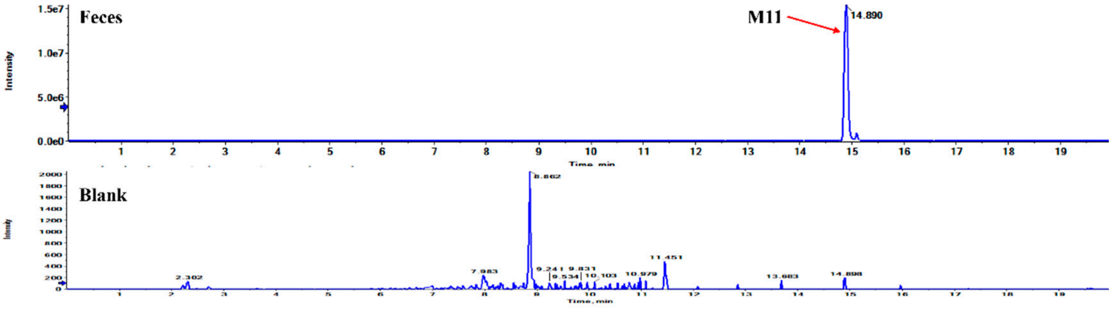


Fig.S12 EIC spectra of metabolites M11 in feces

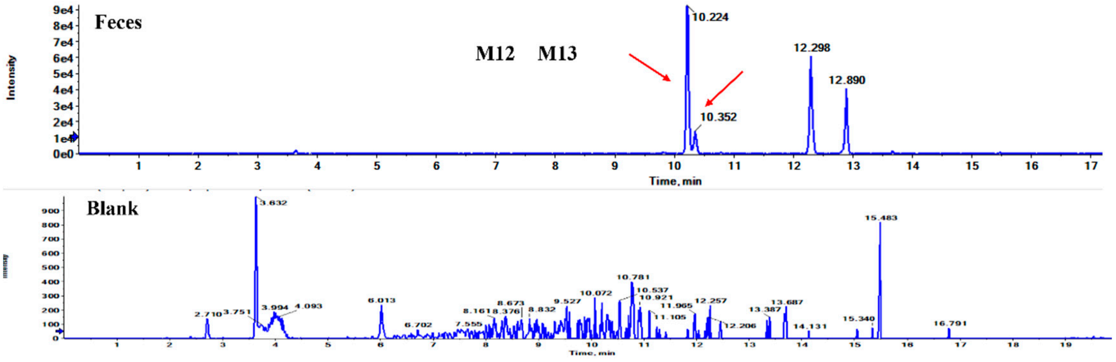


Fig.S13 EIC spectra of metabolites M12 and M13 in feces

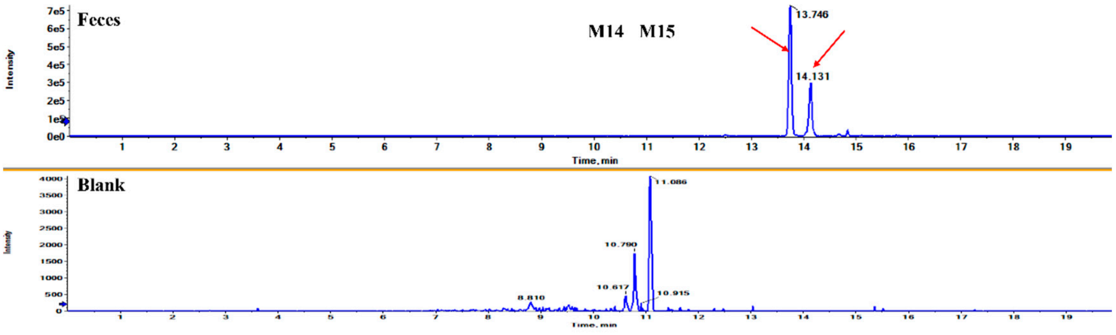


Fig.S14 EIC spectra of metabolites M14 and M15 in feces

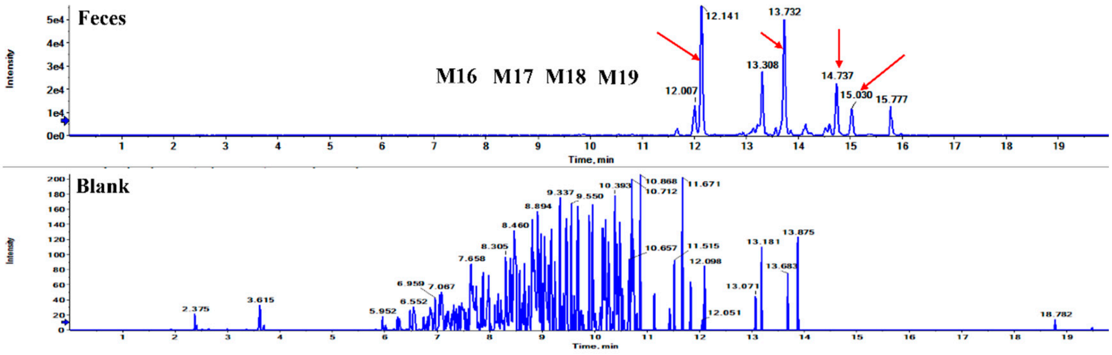


Fig.S15 EIC spectra of metabolites M16–M18 in feces

3. The MS/MS spectrum of metabolites

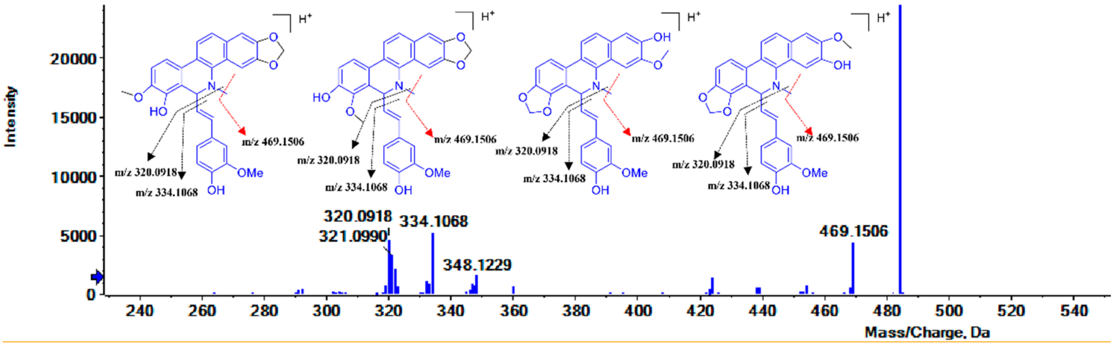


Fig.S16 MS/MS spectrum of M1–M4

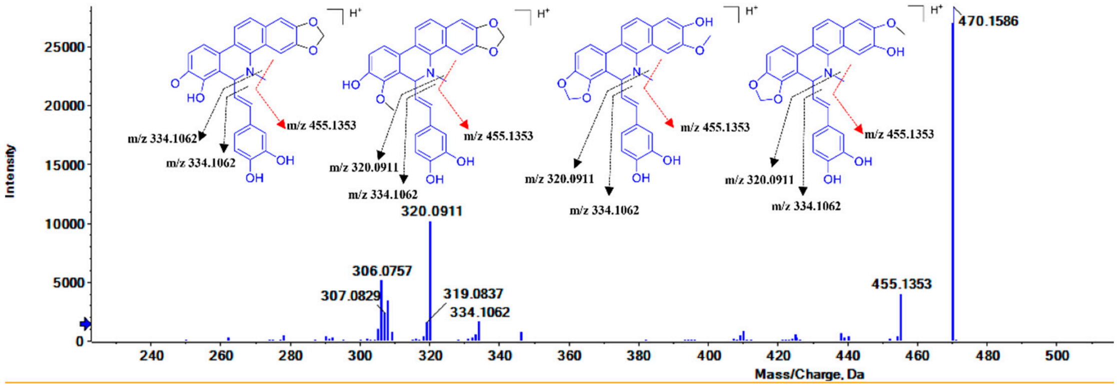


Fig.S17 MS/MS spectrum of M5–M8

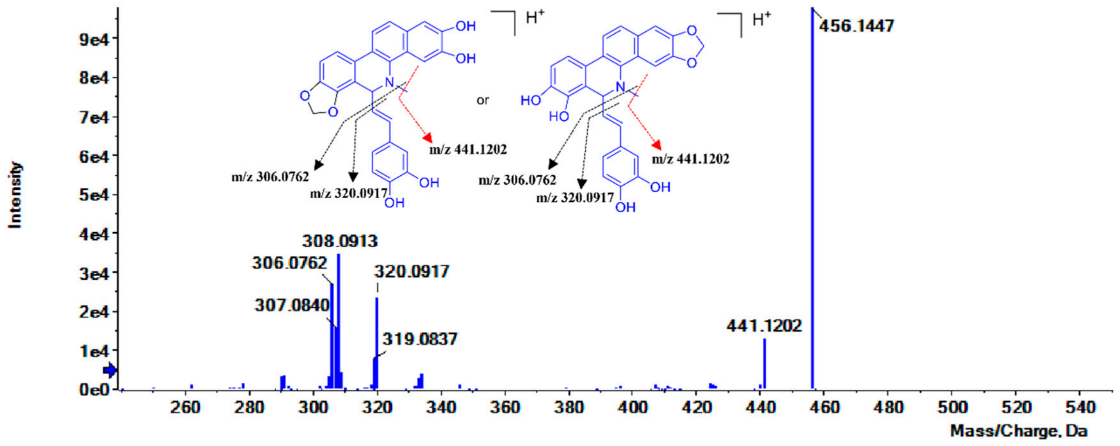


Fig.S18 MS/MS spectrum of M9

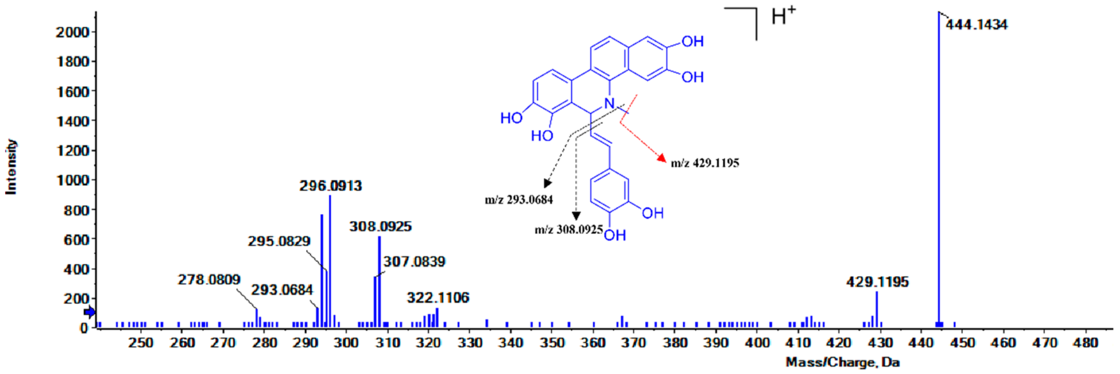


Fig.S19 MS/MS spectrum of M10

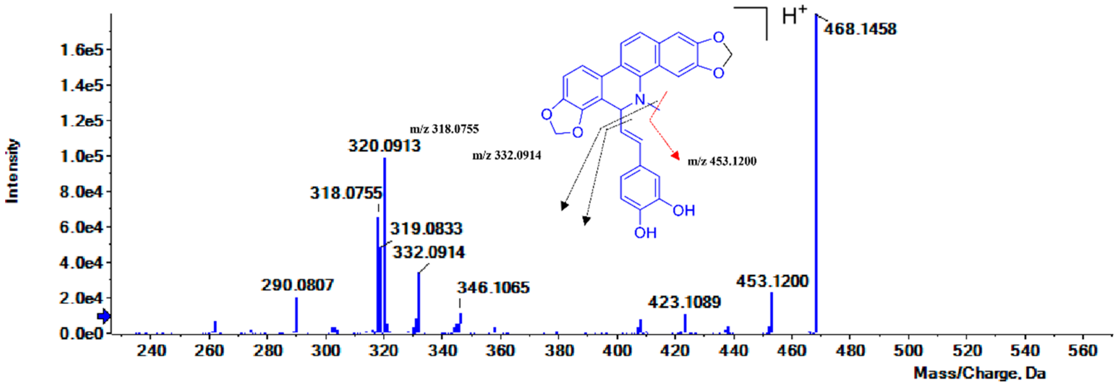


Fig.S20 MS/MS spectrum of M11

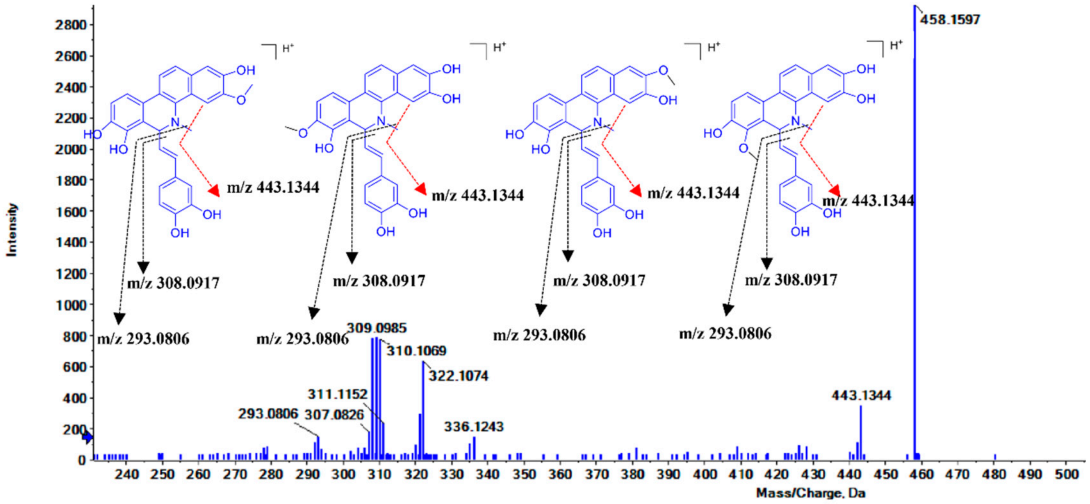


Fig.S21 MS/MS spectrum of M12 and M13

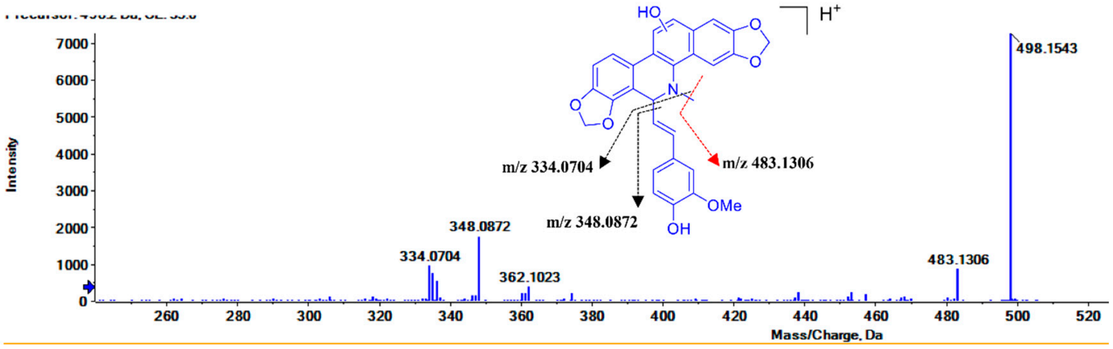


Fig.S22 MS/MS spectrum of M14 and M15

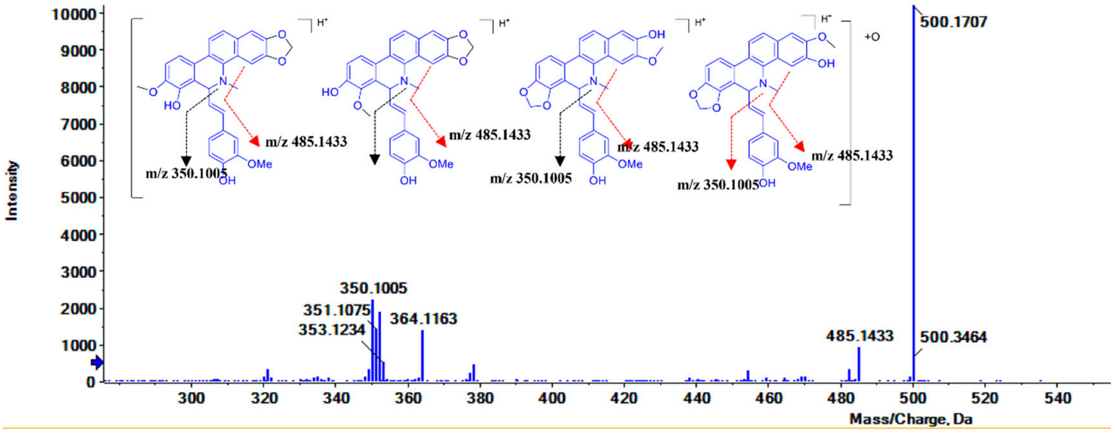


Fig.S23 MS/MS spectrum of M16–M19
