# Supplementary figures and images for: Biotransformation of Maclekarpine E in Rats: CYP2C19-Mediated Metabolism, Fecal Enrichment, and Network Pharmacology-Based Anti-Ulcerative Colitis Prediction
Source: Curr Issues Mol Biol. 2026 Mar 23;48(3):335. doi: 10.3390/cimb48030335 (PMC13024927; doi:10.3390/cimb48030335)

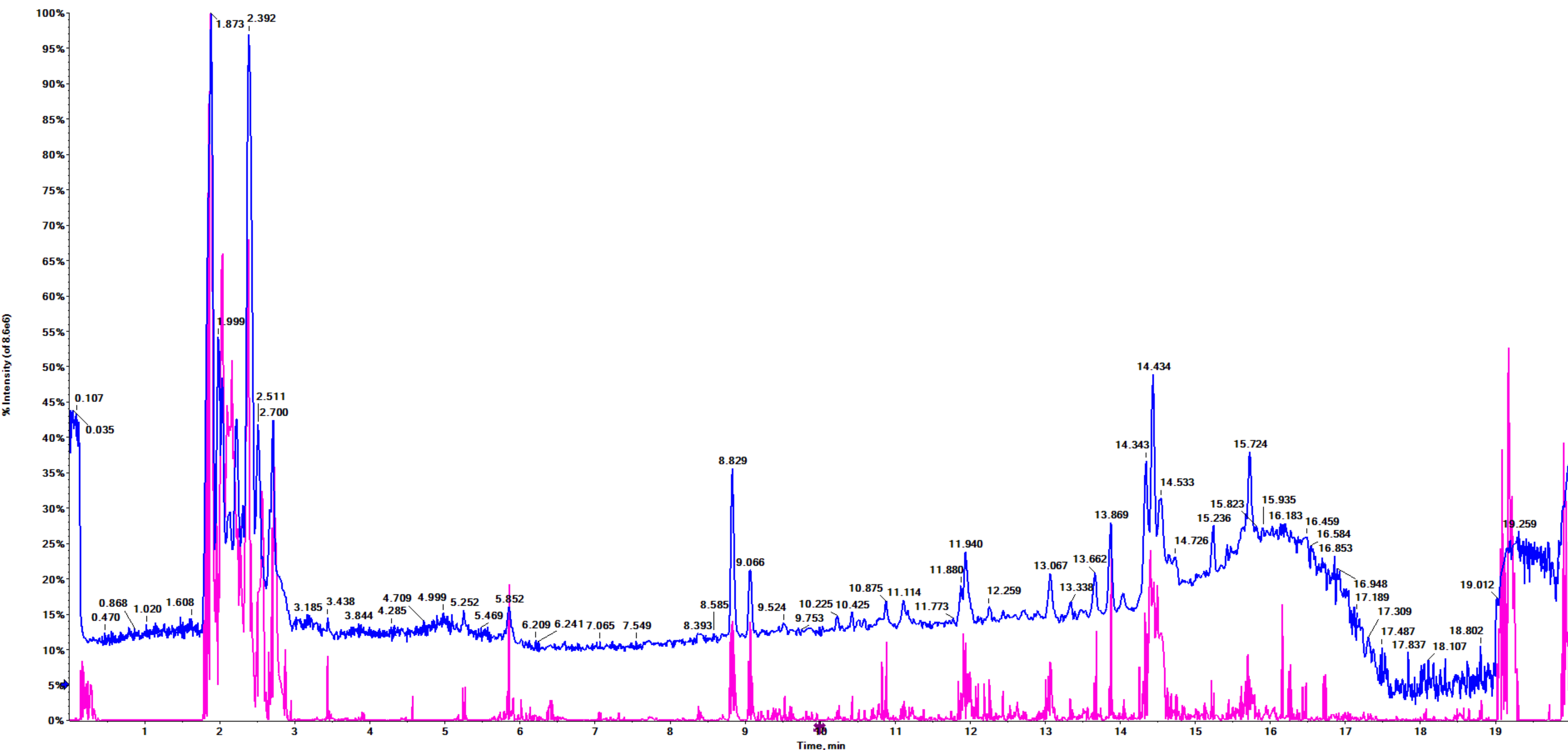

Supplement: Supplementary file 1 [file cimb-48-00335-s001.zip › Fig.S1 TIC spectrum in plasma.tif]

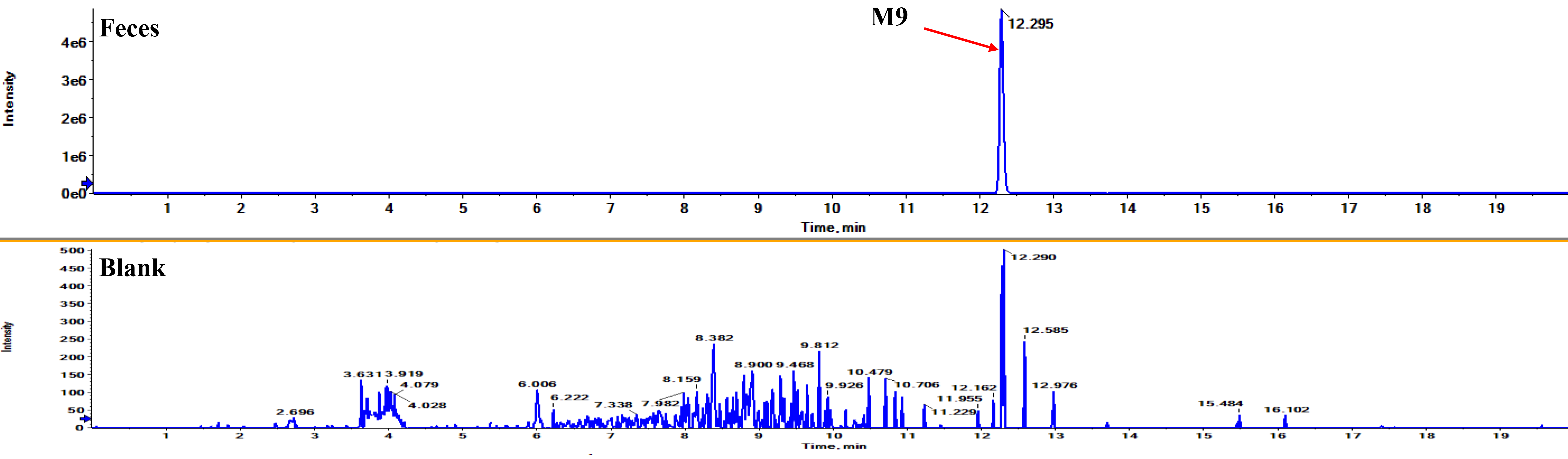

Supplement: Supplementary file 1 [file cimb-48-00335-s001.zip › Fig.S10 EIC spectra of metabolites M9 in feces.tif]

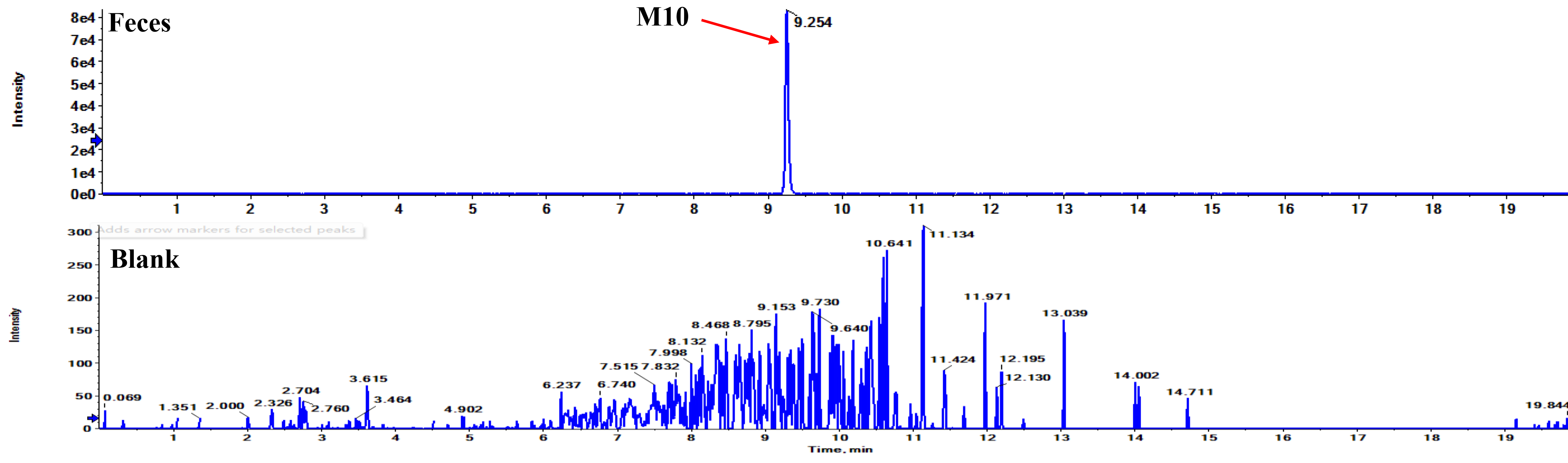

Supplement: Supplementary file 1 [file cimb-48-00335-s001.zip › Fig.S11 EIC spectra of metabolites M10 in feces.tif]

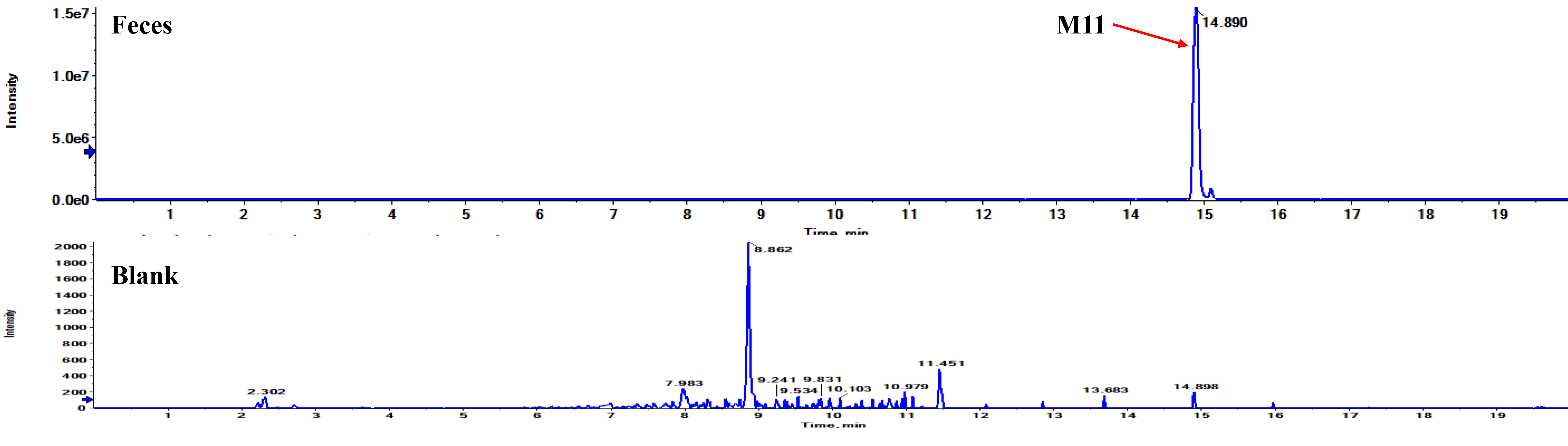

Supplement: Supplementary file 1 [file cimb-48-00335-s001.zip › Fig.S12 EIC spectra of metabolites M11 in feces.tif]

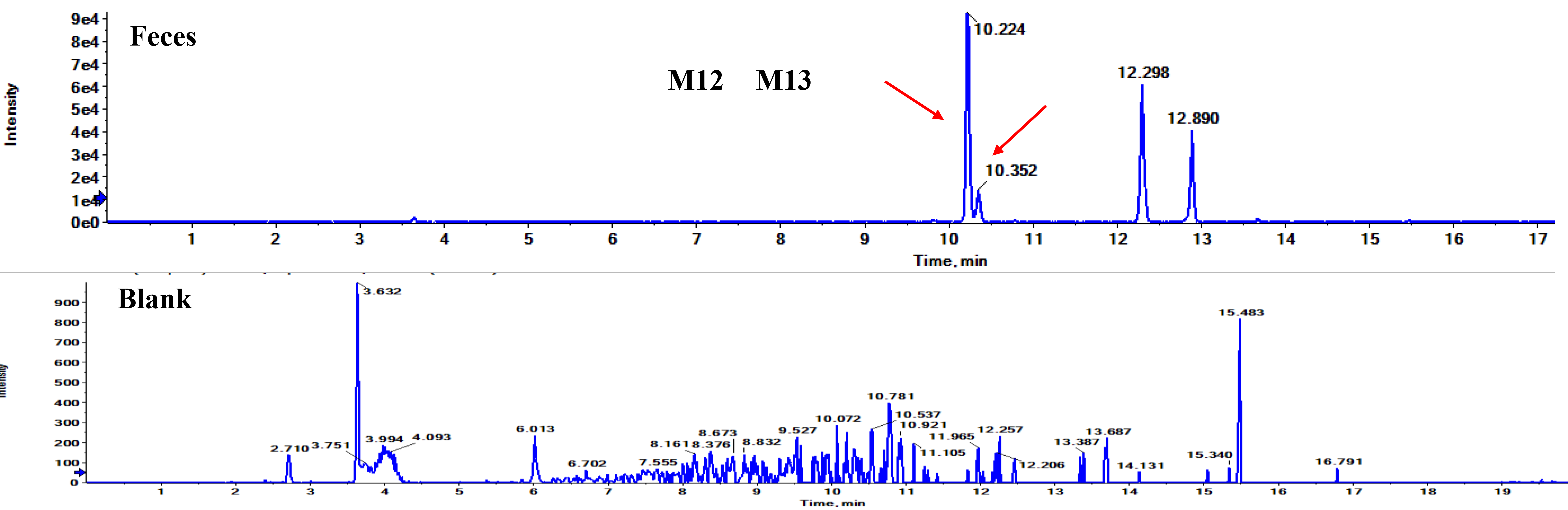

Supplement: Supplementary file 1 [file cimb-48-00335-s001.zip › Fig.S13 EIC spectra of metabolites M12 and M13 in feces.tif]

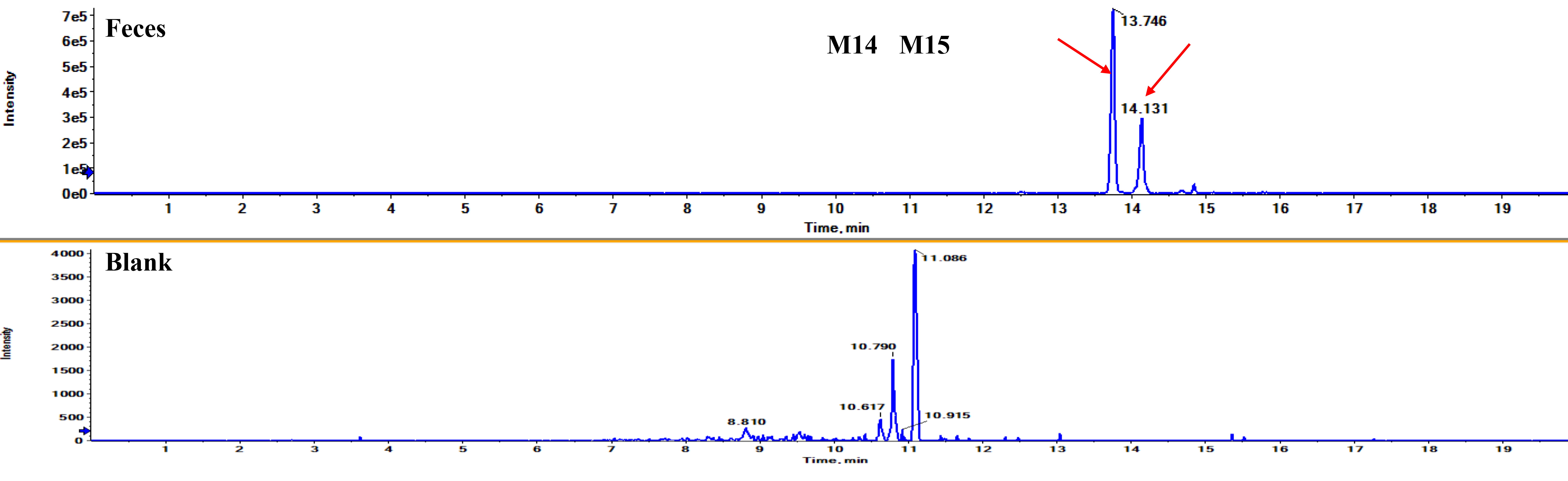

Supplement: Supplementary file 1 [file cimb-48-00335-s001.zip › Fig.S14 EIC spectra of metabolites M14 and M15 in feces.tif]

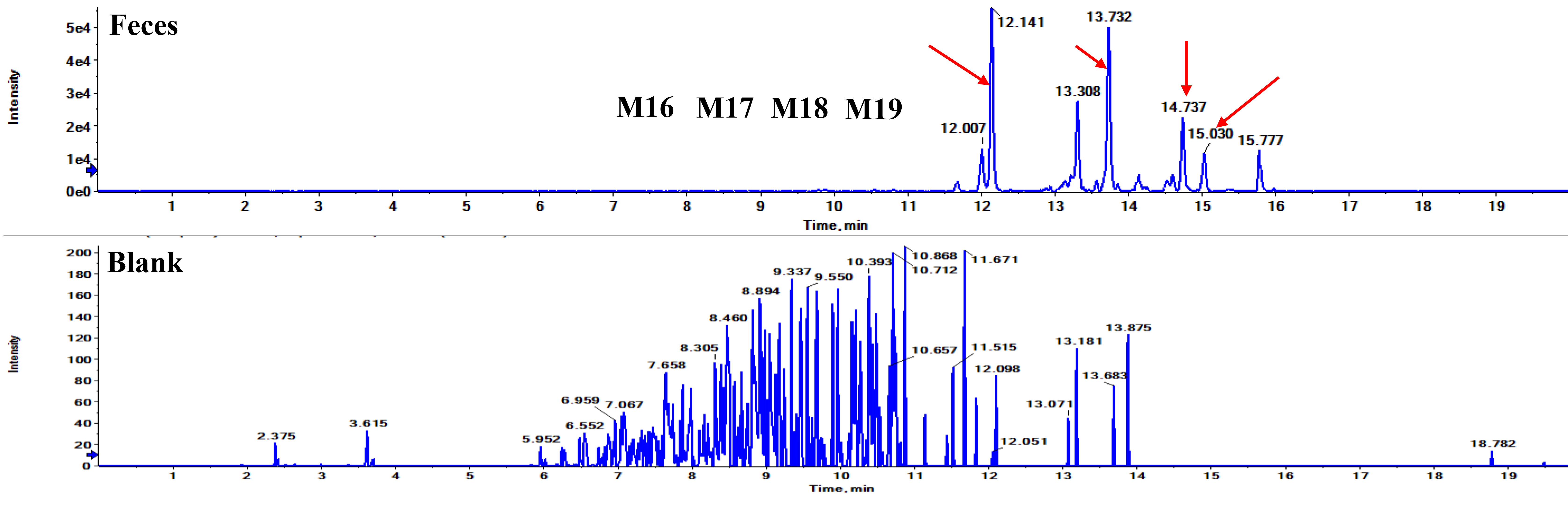

Supplement: Supplementary file 1 [file cimb-48-00335-s001.zip › Fig.S15 EIC spectra of metabolites M16¿CM18 in feces.tif]

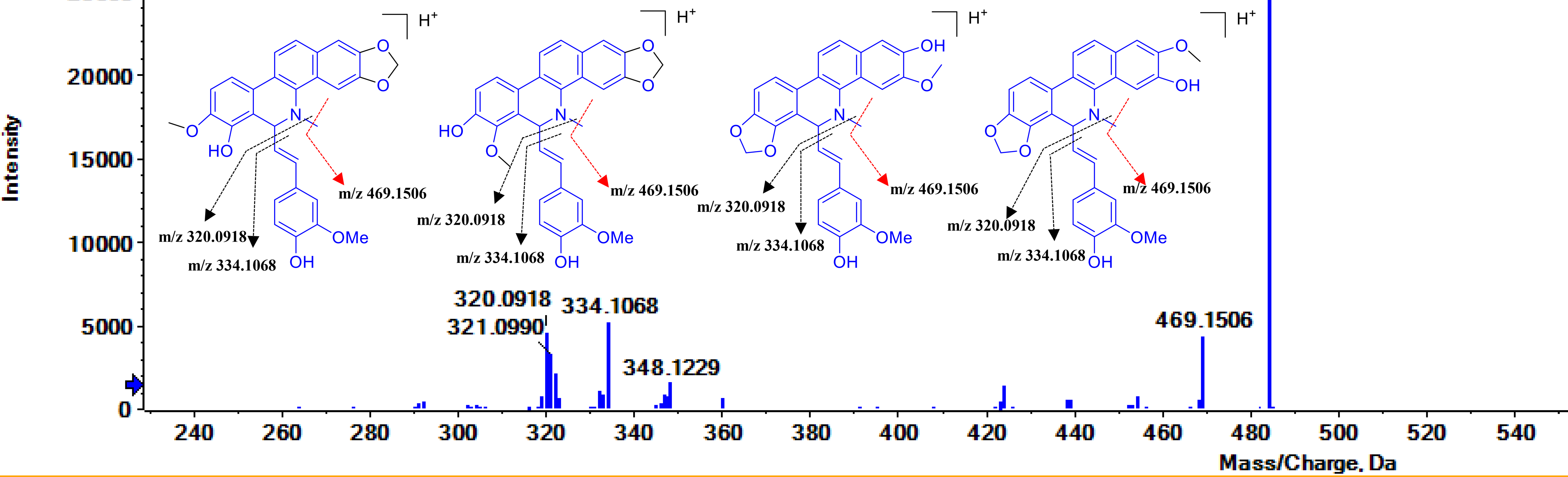

Supplement: Supplementary file 1 [file cimb-48-00335-s001.zip › Fig.S16 MSMS spectrum of M1¿CM4.tif]

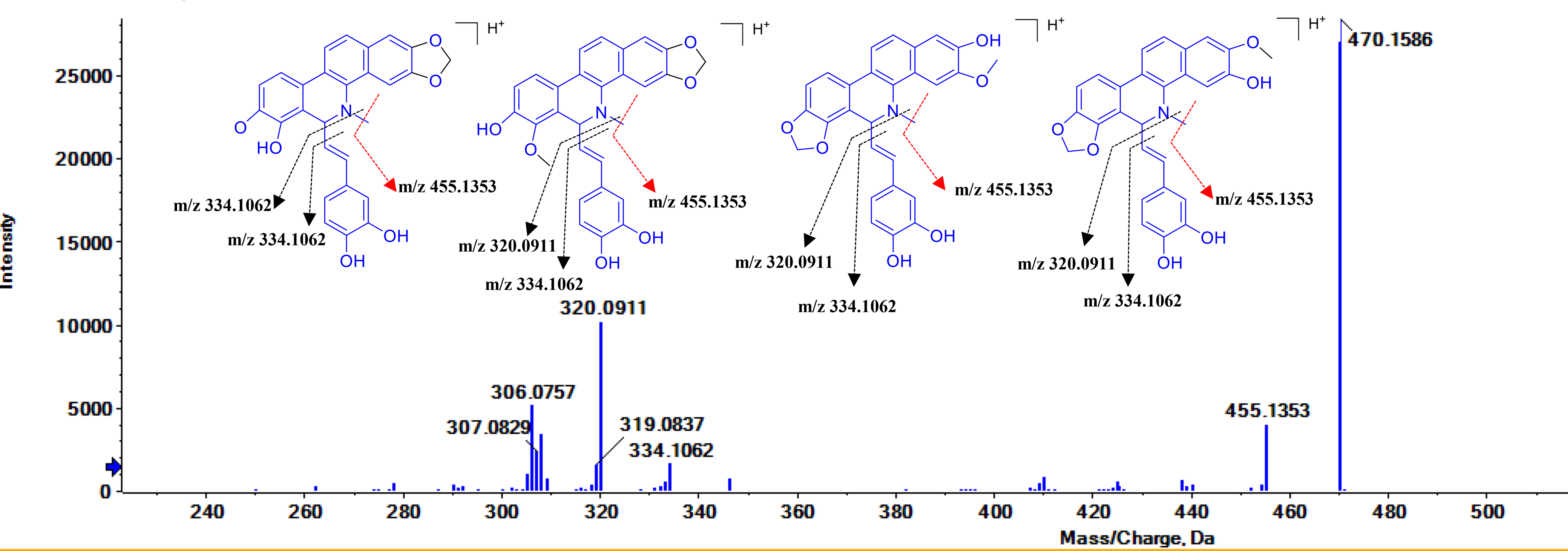

Supplement: Supplementary file 1 [file cimb-48-00335-s001.zip › Fig.S17 MSMS spectrum of M5¿CM8.tif]

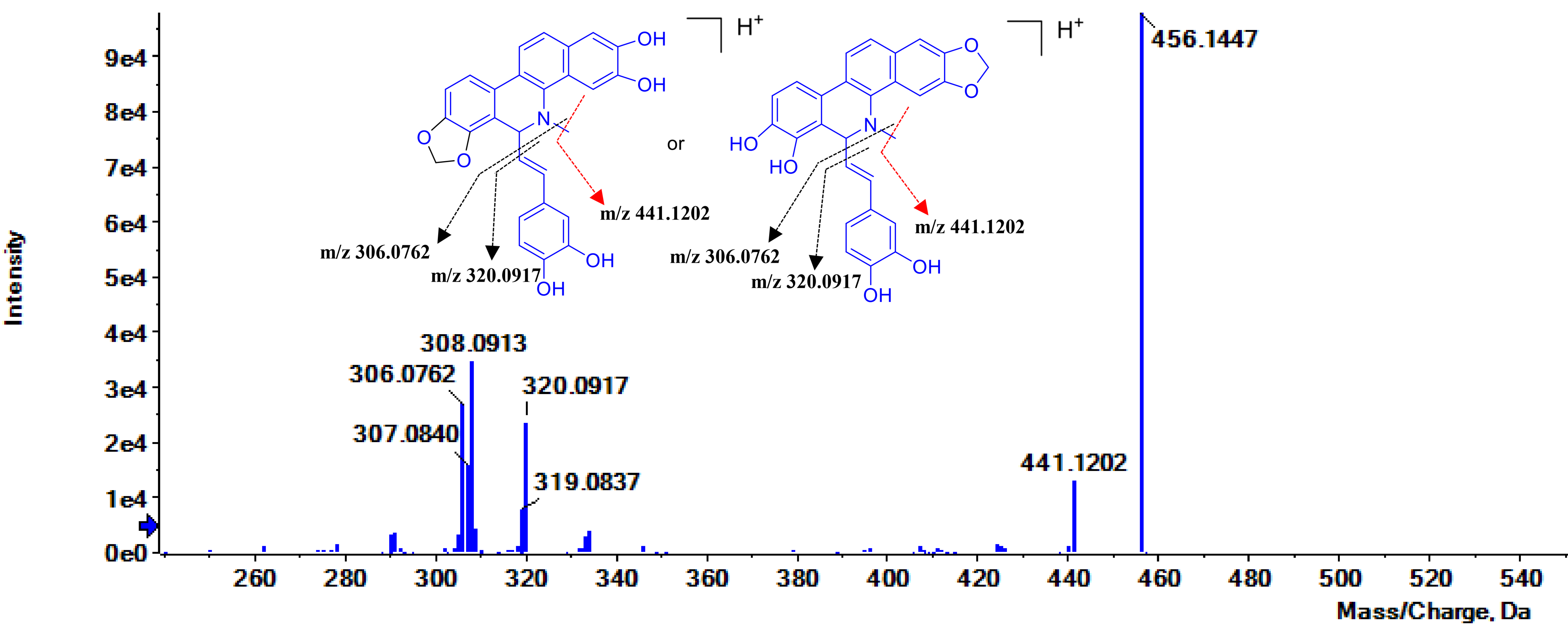

Supplement: Supplementary file 1 [file cimb-48-00335-s001.zip › Fig.S18 MSMS spectrum of M9.tif]

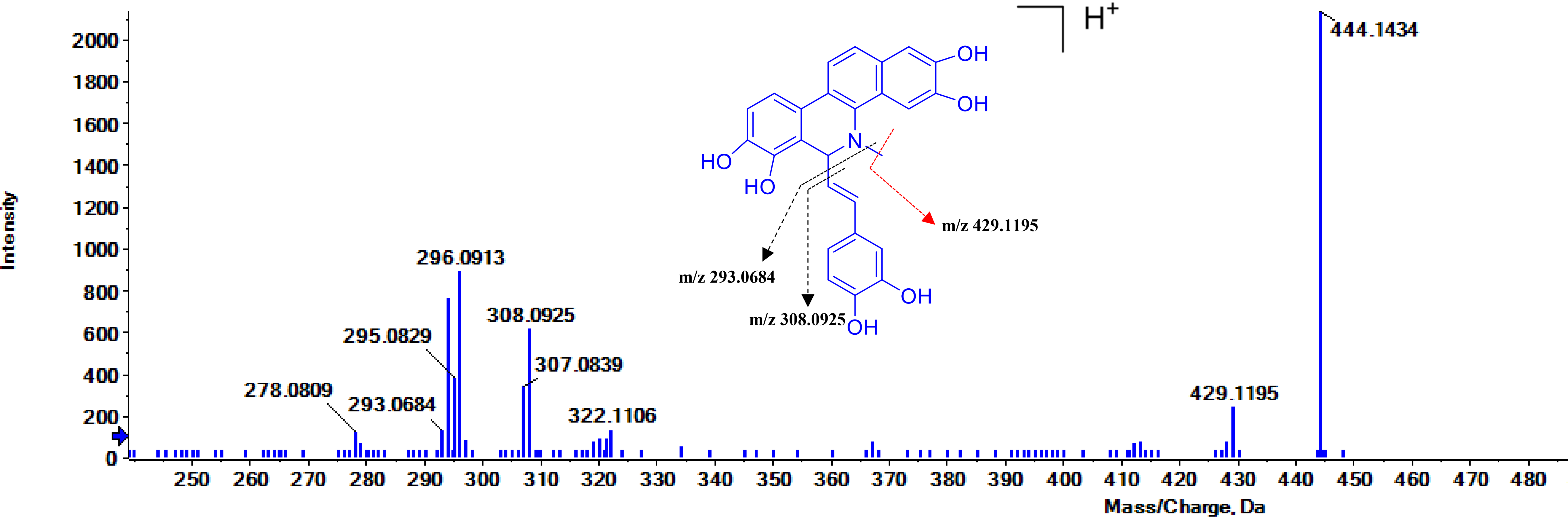

Supplement: Supplementary file 1 [file cimb-48-00335-s001.zip › Fig.S19 MSMS spectrum of M10.tif]

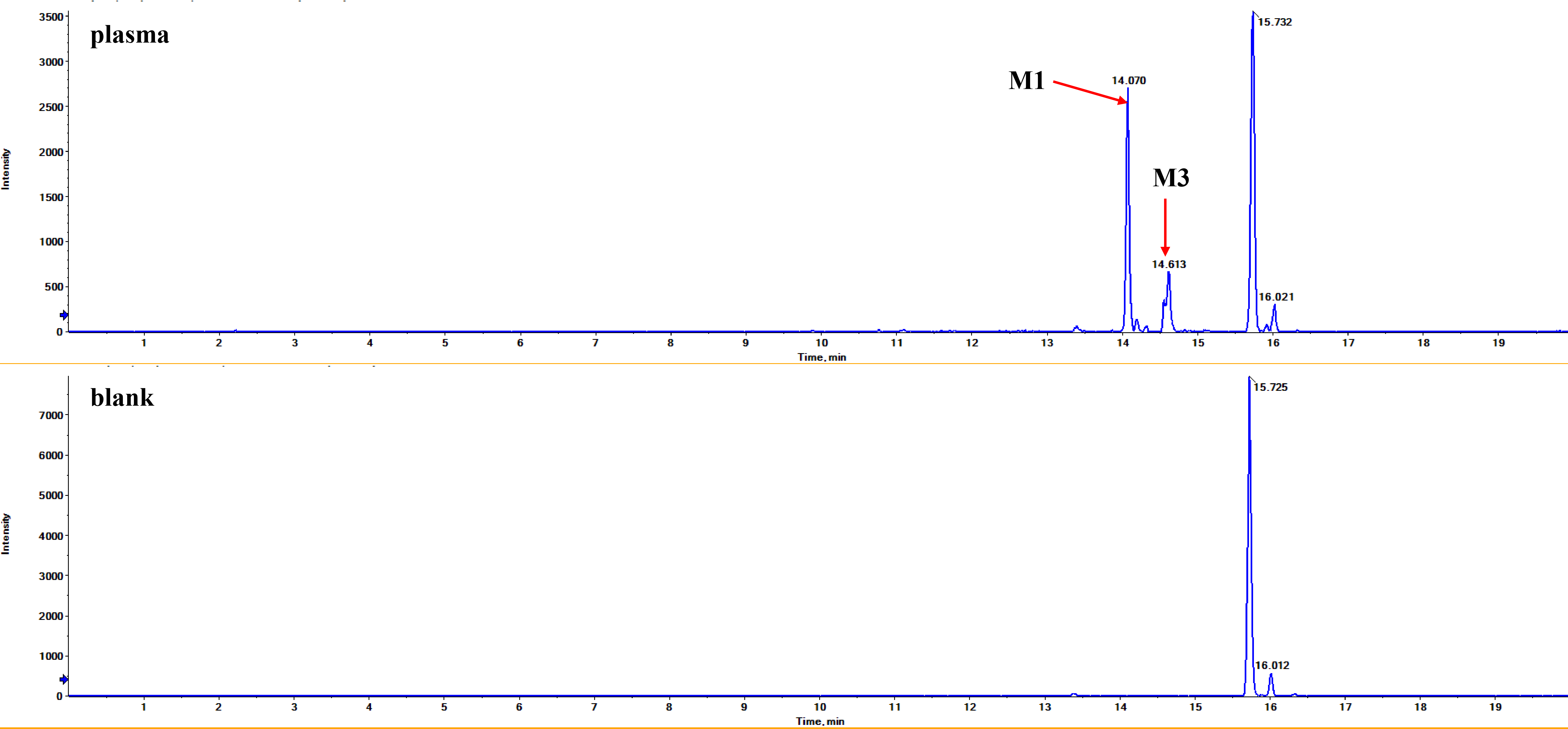

Supplement: Supplementary file 1 [file cimb-48-00335-s001.zip › Fig.S2 EIC spectra of metabolites M1 and M3.tif]

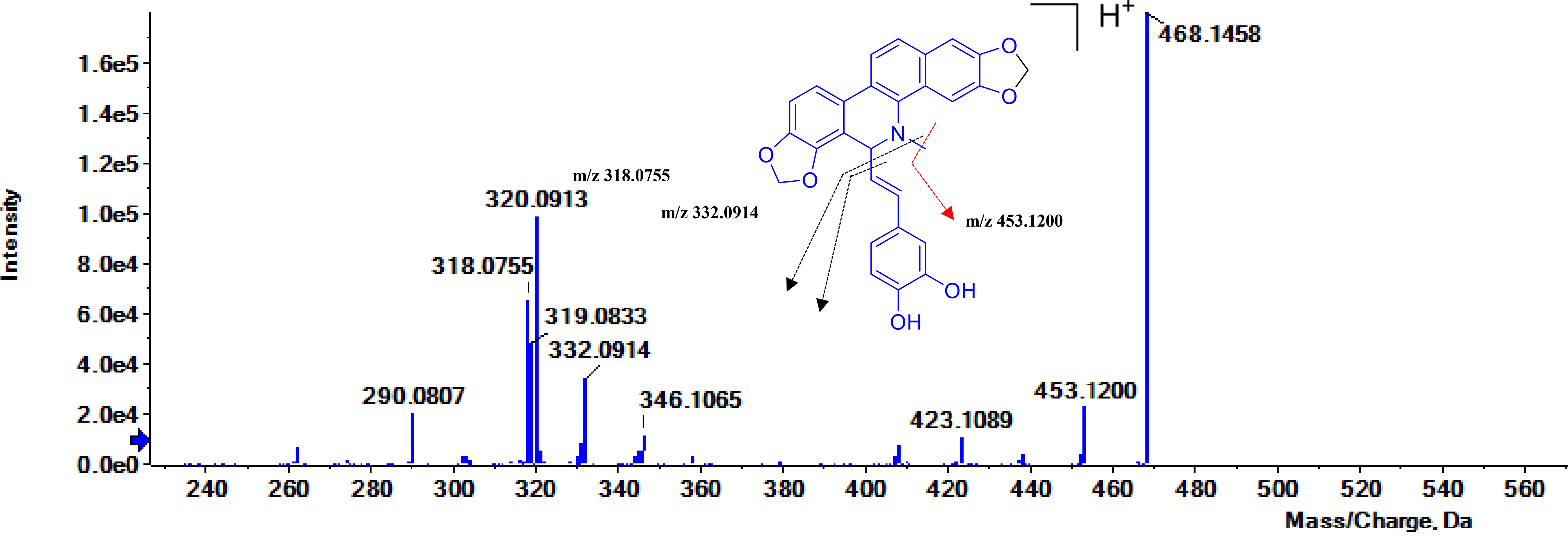

Supplement: Supplementary file 1 [file cimb-48-00335-s001.zip › Fig.S20 MSMS spectrum of M11.tif]

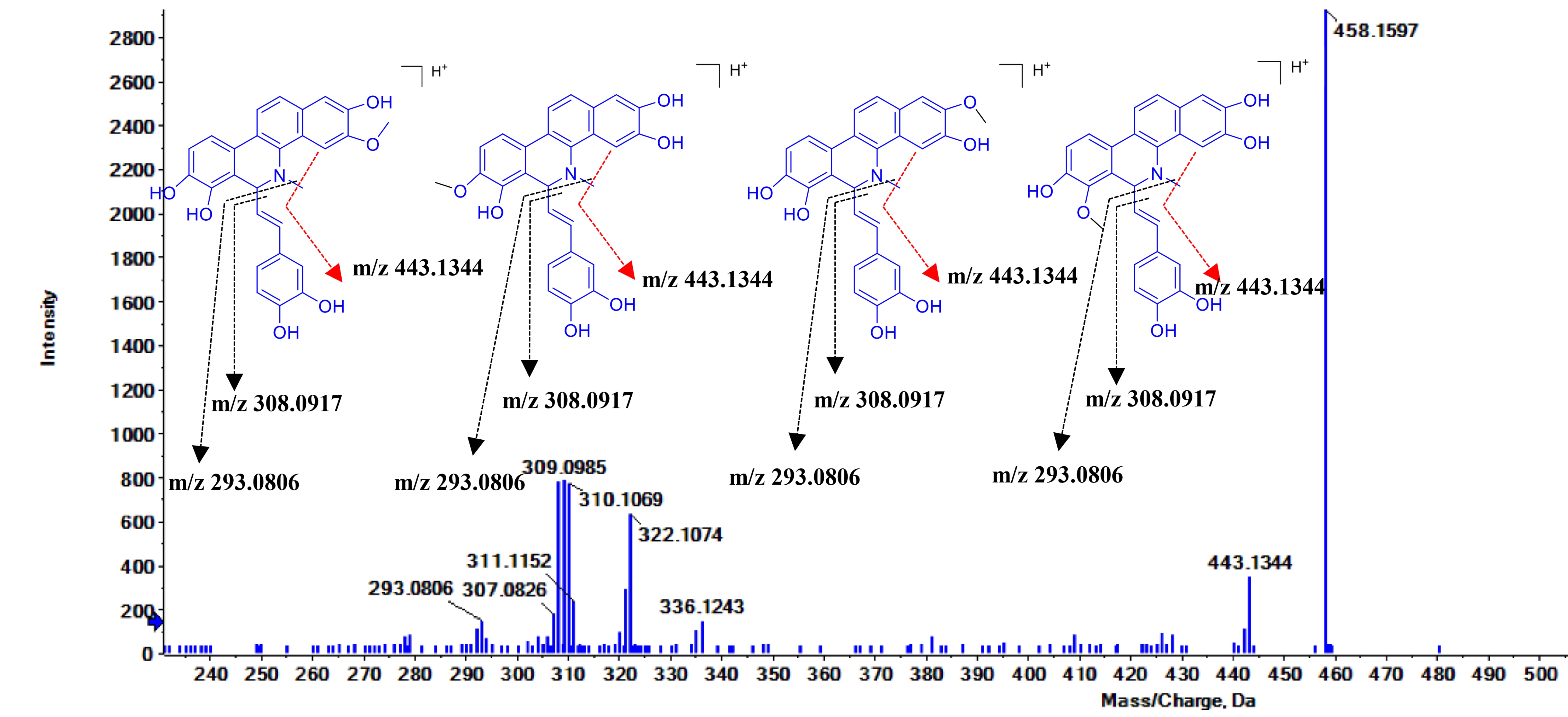

Supplement: Supplementary file 1 [file cimb-48-00335-s001.zip › Fig.S21 MSMS spectrum of M12 and M13.tif]

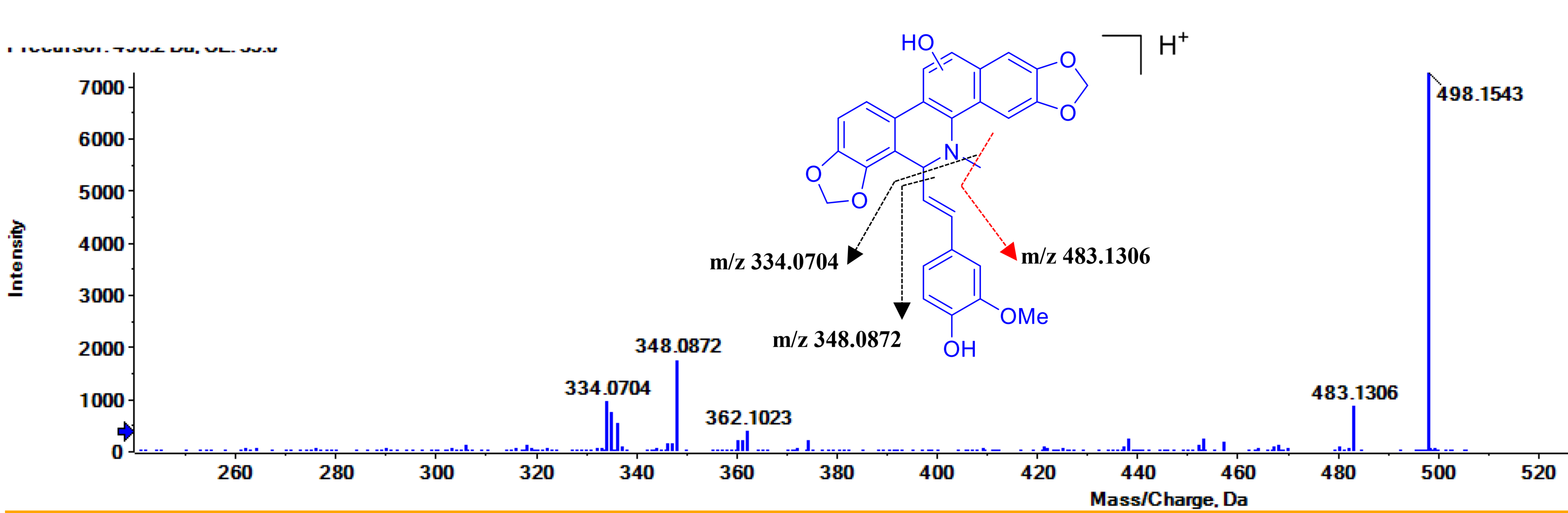

Supplement: Supplementary file 1 [file cimb-48-00335-s001.zip › Fig.S22 MSMS spectrum of M14 and M15.tif]

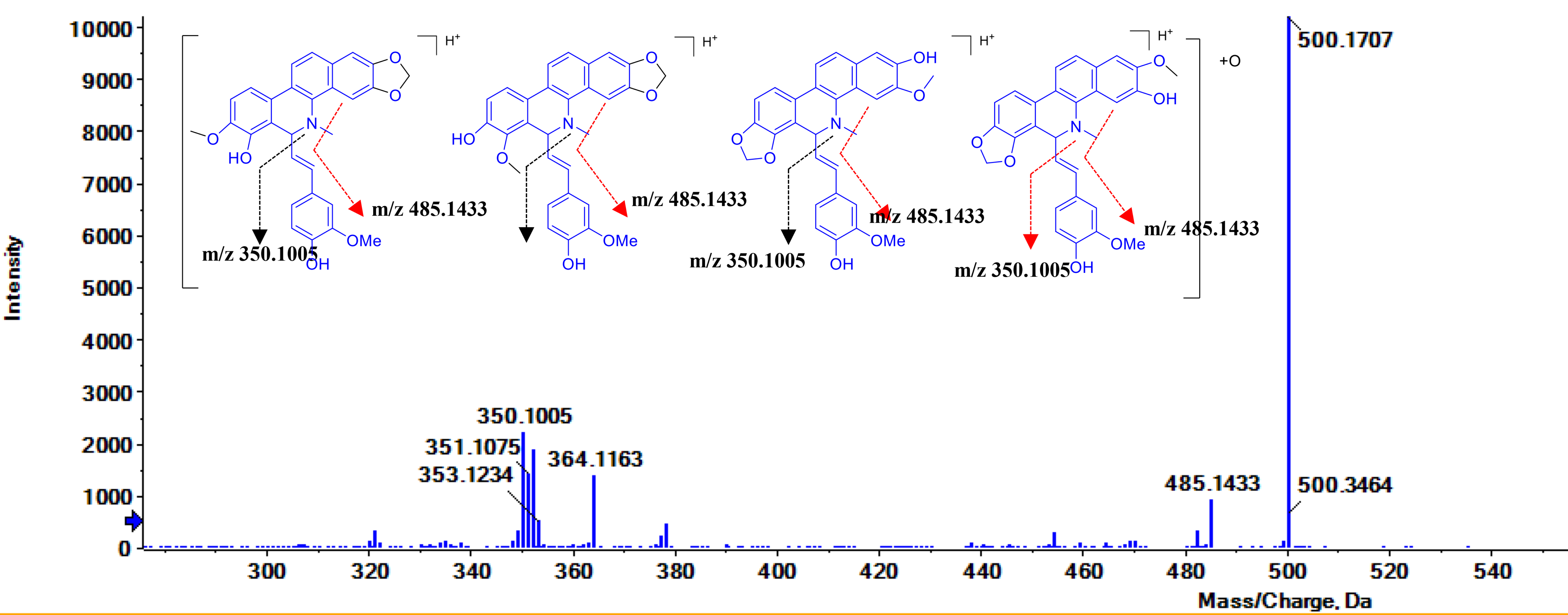

Supplement: Supplementary file 1 [file cimb-48-00335-s001.zip › Fig.S23 MSMS spectrum of M16¿CM19.tif]

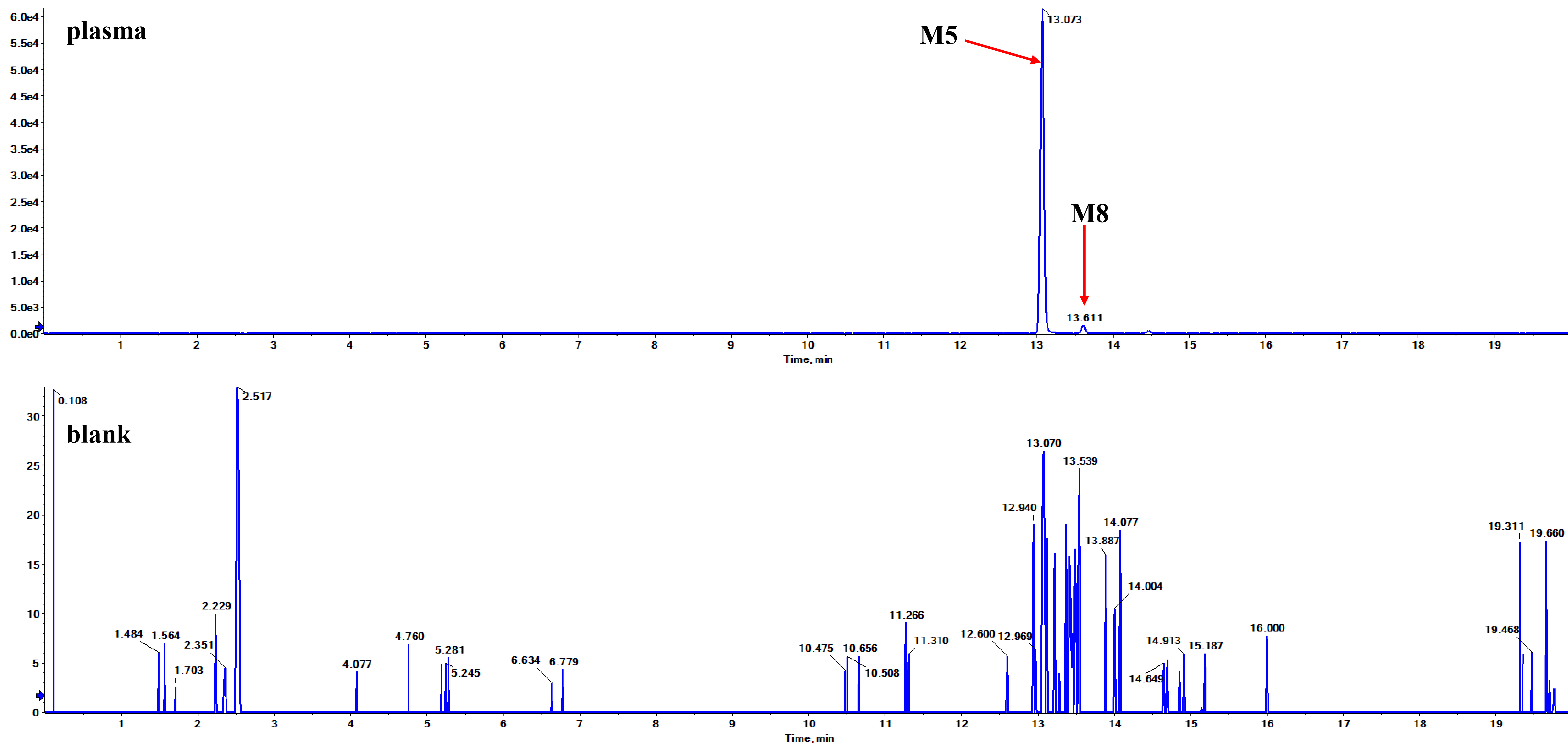

Supplement: Supplementary file 1 [file cimb-48-00335-s001.zip › Fig.S3 EIC spectra of metabolites M5 and M8.tif]

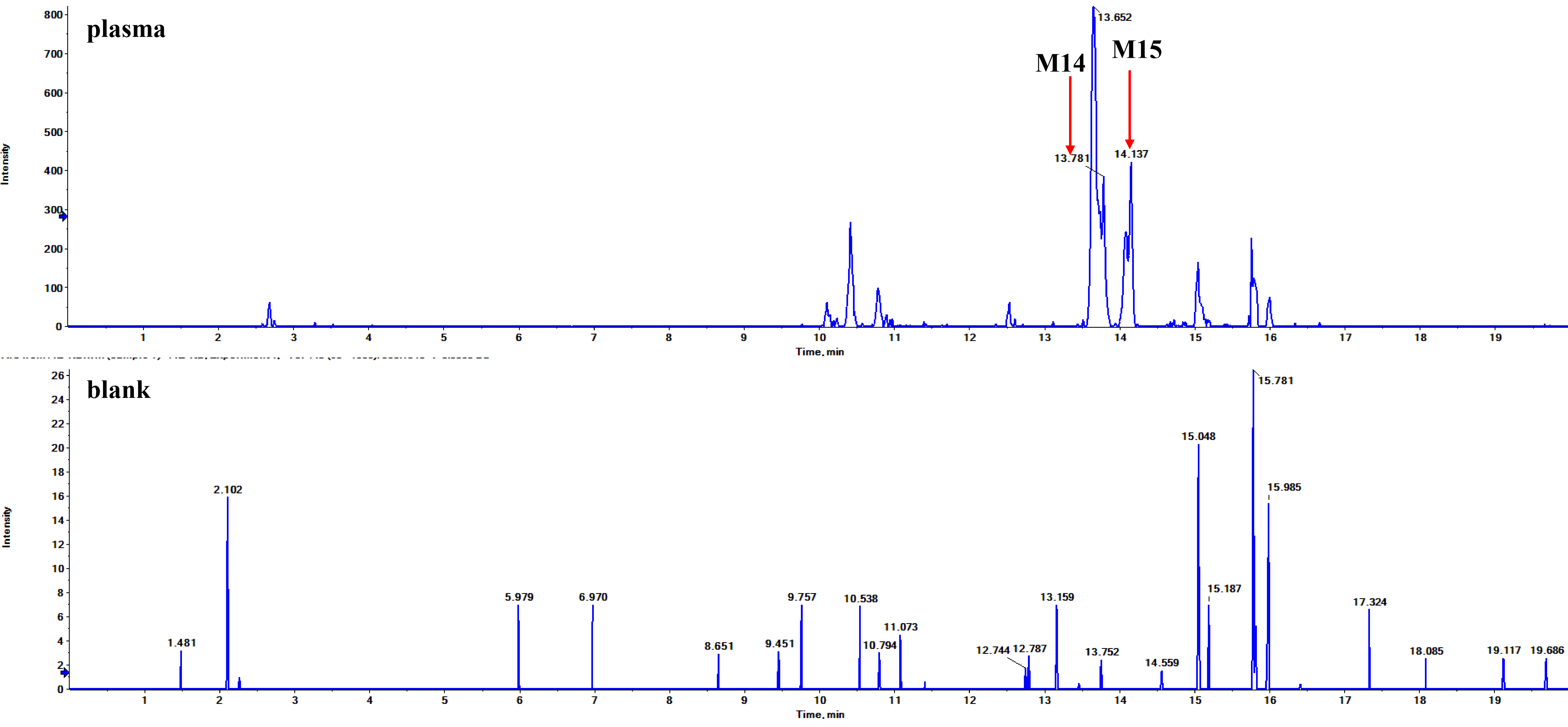

Supplement: Supplementary file 1 [file cimb-48-00335-s001.zip › Fig.S4 EIC spectra of metabolites M14 and M15.tif]

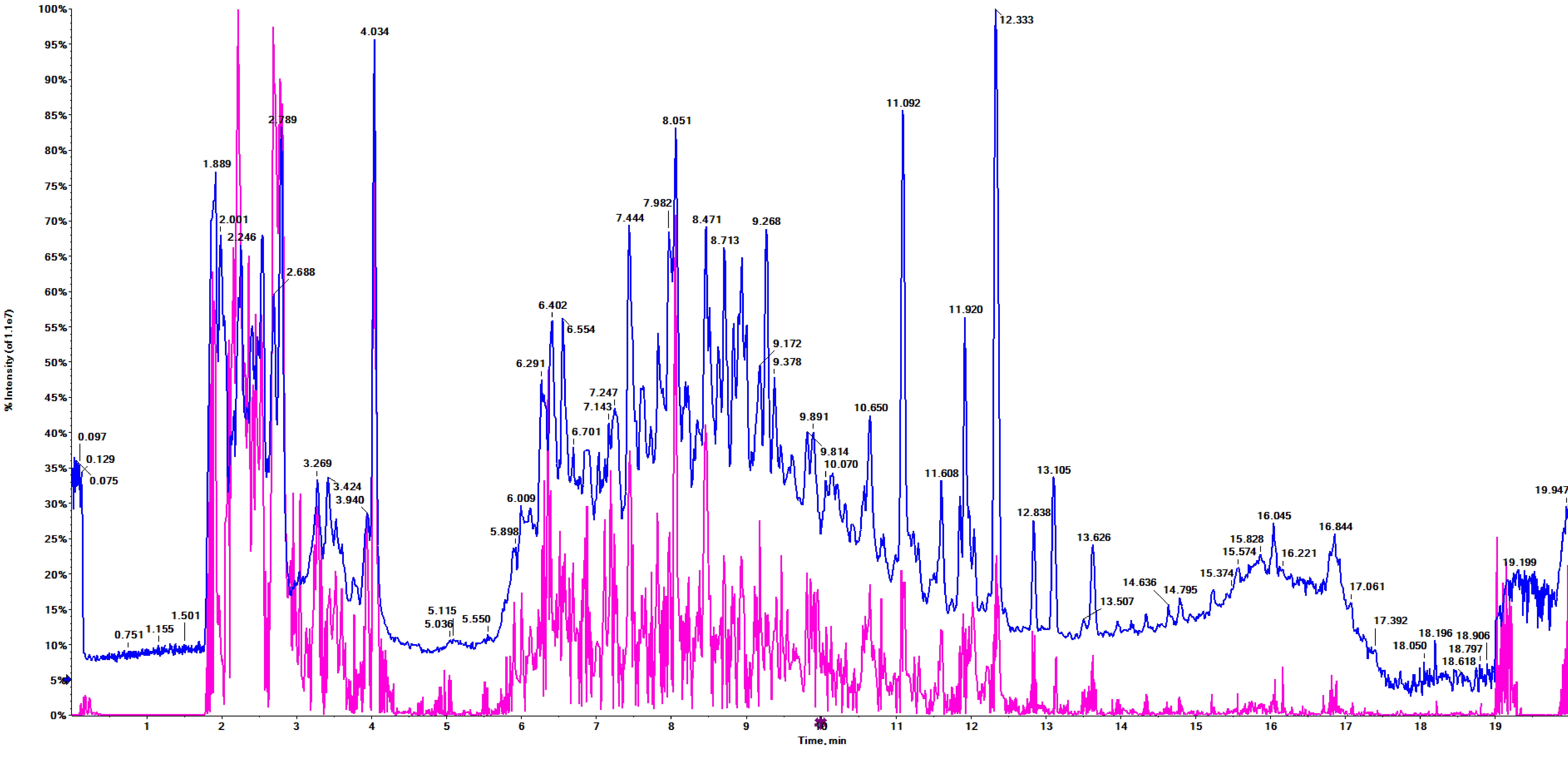

Supplement: Supplementary file 1 [file cimb-48-00335-s001.zip › Fig.S5 TIC spectrum in urine.tif]

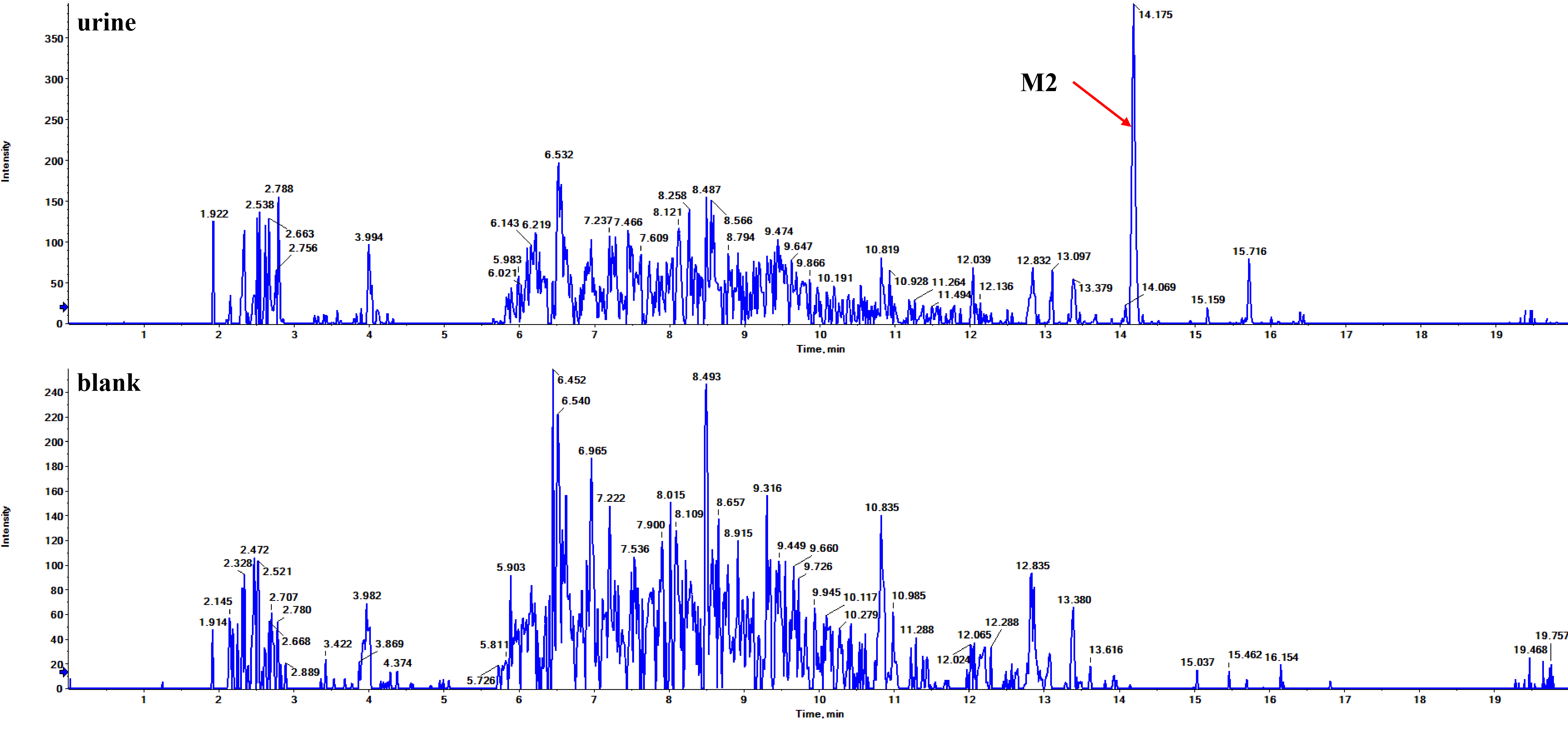

Supplement: Supplementary file 1 [file cimb-48-00335-s001.zip › Fig.S6 EIC spectra of metabolites M12 in urine.tif]

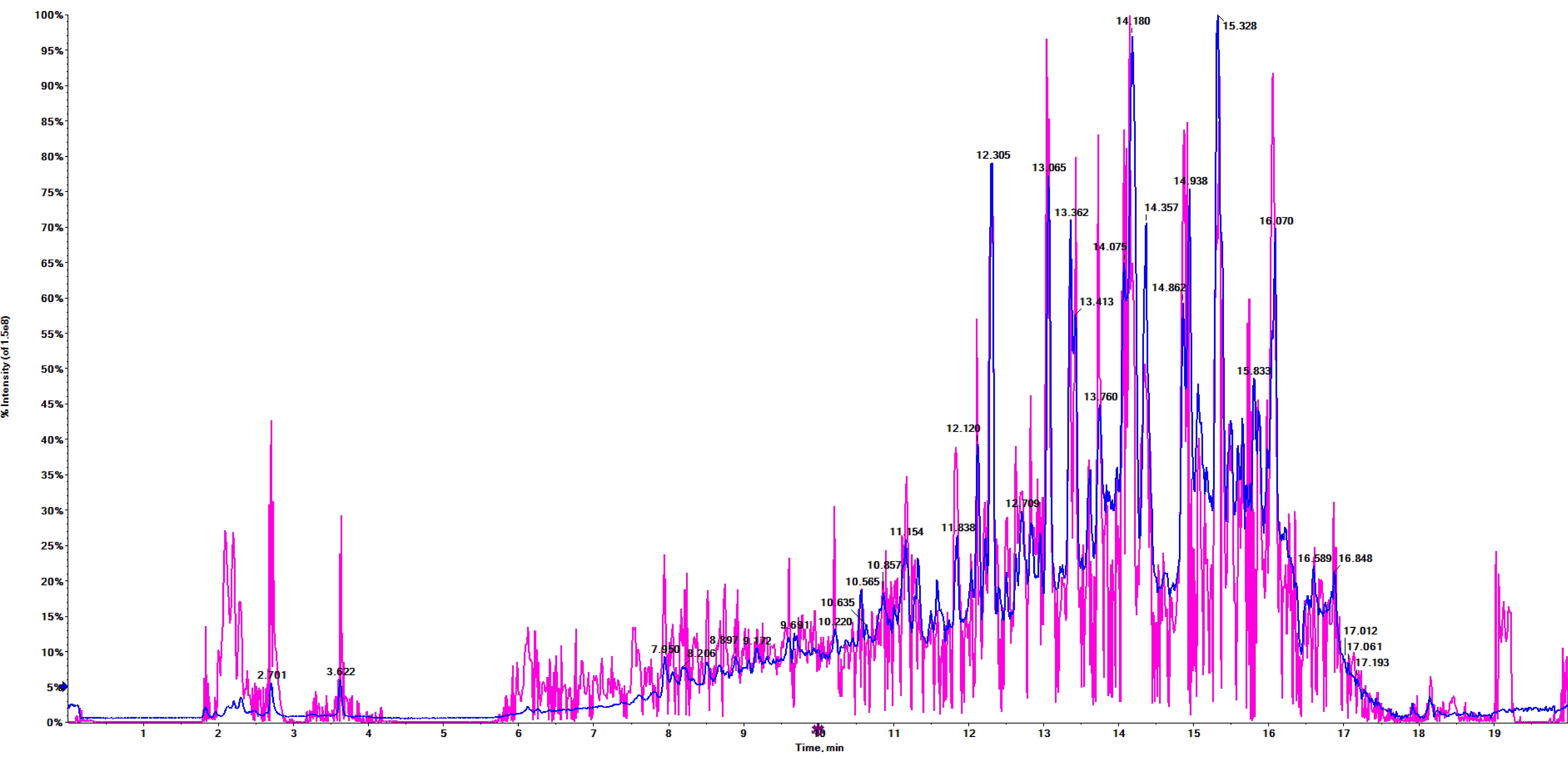

Supplement: Supplementary file 1 [file cimb-48-00335-s001.zip › Fig.S7 TIC spectrum in feces.tif]

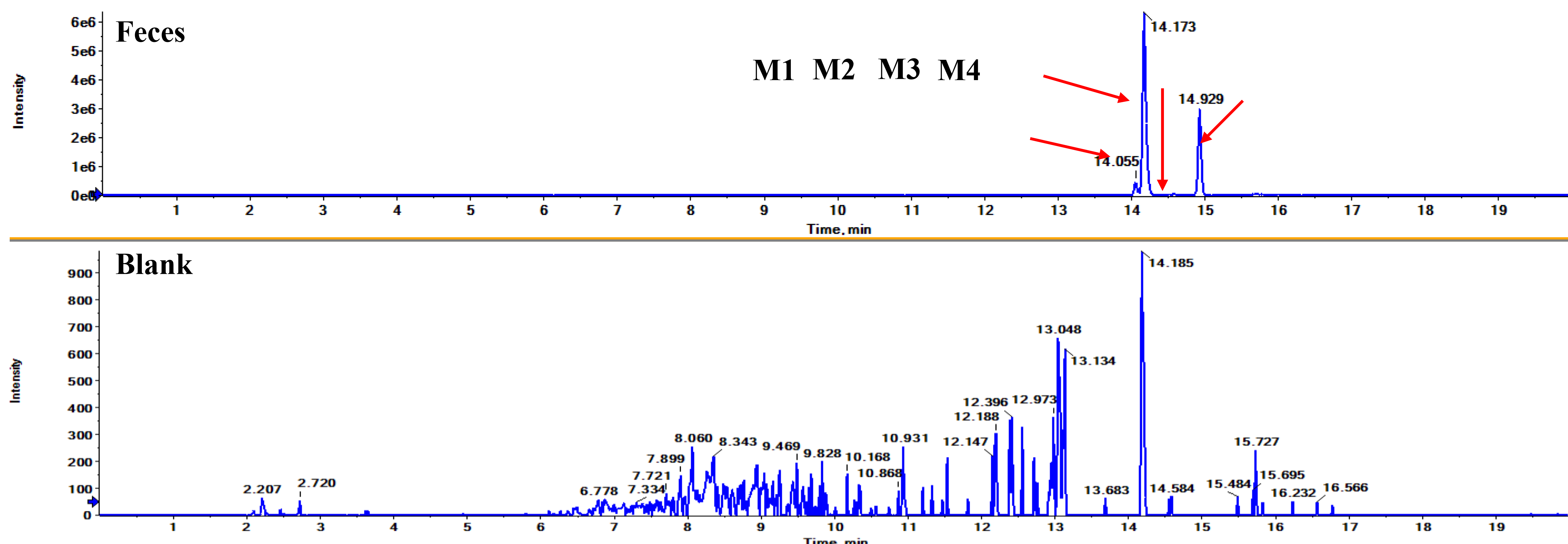

Supplement: Supplementary file 1 [file cimb-48-00335-s001.zip › Fig.S8 EIC spectra of metabolites M1¿CM4 in feces.tif]

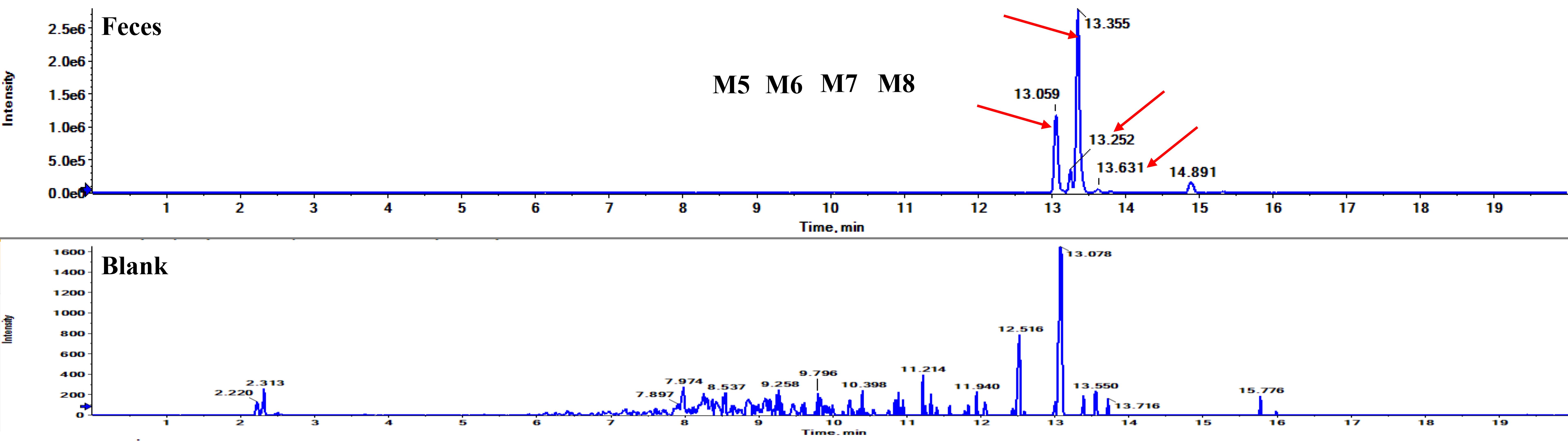

Supplement: Supplementary file 1 [file cimb-48-00335-s001.zip › Fig.S9 EIC spectra of metabolites M5¿CM8 in feces.tif]
